# Exploration of the biodiversity and mining novel target genes of *Listeria monocytogenes* strains isolated from beef through comparative genomics analysis

**DOI:** 10.3389/fmicb.2025.1560974

**Published:** 2025-04-28

**Authors:** Bo Zhang, Wenjie Sun, Xiaoxu Wang, Honglin Ren, Yang Wang, Shaohui Hu, Chengwei Li, Yuzhu Wang, Jiaqi Hou, Xueyu Hu, Ruoran Shi, Yansong Li, Shiying Lu, Qiang Lu, Zengshan Liu, Pan Hu

**Affiliations:** ^1^State Key Laboratory for Diagnosis and Treatment of Severe Zoonotic Infectious Diseases, Key Laboratory for Zoonosis Research of the Ministry of Education, Institute of Zoonosis, and College of Veterinary Medicine, Jilin University, Changchun, China; ^2^Institute of Special Animal and Plant Sciences of Chinese Academy of Agricultural Sciences, Changchun, China

**Keywords:** *Listeria monocytogenes*, comparative genomics, pan-genomics, genetic evaluations, target genes

## Abstract

*L. monocytogenes* is a significant foodborne pathogen. This study aims to explore the biodiversity and evolutionary characteristics of *L. monocytogenes* isolated from beef through pan-genome analysis, and to provide important reference value for its specific molecular detection. This study conducted an in-depth analysis of the virulence genes, antimicrobial resistance genes, and environmental resistance genes of 344 *L. monocytogenes* strains isolated from beef. Pan-genomic analysis revealed that *L. monocytogenes* from beef have open genomes, providing a solid genetic basis for adaptation to different environments. MLST analysis revealed that the most prevalent types of *L. monocytogenes* isolated from beef were ST9 and CC9. A total of 50 virulence genes were detected in these strains, with 26 virulence genes such as *inlA*, *inlB*, *plcA*, *plcB*, and *prfA*, present in all *L. monocytogenes* strains. The four most prevalent antibiotic resistance genes in *L. monocytogenes* were *norB*, *lin*, *mprF*, and *FosX*, indicating high resistance to fluoroquinolones, lincosamides, peptides, and phosphonic acid antibiotics. A total of 416 potential target genes were identified through pan-genomic screening, which were then further filtered using a hub gene selection method to mining novel target genes. Ultimately, 10 highly connected hub genes were selected: *bglF_2*, *tilS*, *group_2105*, *group_2431*, *oleD*, *ndk*, *flgG*, *purB*, *pbpB*, and *fni*. These genes play a crucial role in the pathogenesis of *L. monocytogenes*. The PCR results demonstrated the excellent specificity of the *bglF_2* gene for *L. monocytogenes*. Moreover, in the artificial contamination experiment, the *bglF_2* gene was able to effectively detect *L. monocytogenes* in beef samples. Therefore, the *bglF_2* gene holds potential as a specific molecular target for the detection of *L. monocytogenes* strains in beef samples.

## Introduction

*L. monocytogenes* is a foodborne pathogen belonging to Gram-positive bacteria. This highly adaptable bacterium can thrive under adverse conditions, including low temperatures, high salinity, and extreme pH levels. It is commonly associated with food contamination, particularly in meat products (Zhang et al., 2021; Montero et al., 2015). It can cause listeriosis in humans, particularly in populations with compromised immune function, such as neonates, the elderly, pregnant women, and individuals with weakened immune systems. The onset of the disease may be accompanied by various symptoms, including mild diarrhea, meningitis, and septicemia (Matle et al., 2020; Colomba et al., 2020). Listeriosis is typically caused by the consumption of contaminated food, making food safety particularly important for the prevention and control of the disease.

The detection of *L. monocytogenes* in food typically involves biochemical and molecular methods (Gupta and Adhikari, 2022; Law et al., 2015). Biochemical methods are conventional and accurate, but require a 7-day bacterial incubation period followed by morphological, biochemical, and serological confirmation. This approach is labor-intensive and time-consuming, making it unsuitable for rapid detection (Sloan et al., 2017). Molecular methods, on the other hand, can shorten the detection time to a few hours, allowing for precise and rapid identification of the bacteria (Buszewski et al., 2017; Feng et al., 2020). Molecular techniques developed for *L. monocytogenes* detection include PCR, quantitative PCR (qPCR), and multiplex PCR (Liu, 2013). The *hly* gene is an important virulence factor in the infection process of *L. monocytogenes*. It encodes a toxin called listeriolysin O (LLO), which facilitates the escape and infection of the host by *L. monocytogenes*. As a result, the *hly* gene has been widely used in PCR-based detection for identifying *L. monocytogenes* (Le et al., 2011). However, it is important to note that not all *L. monocytogenes* strains carry the *hly* gene (Dapgh and Salem, 2022; Ata et al., 2017; Li et al., 2021), indicating the need to mining more specific genes for the detection of *L. monocytogenes*.

In recent years, with the widespread application of next-generation sequencing and third-generation sequencing technologies, a large number of *L. monocytogenes* genomes have been sequenced and shared (Lakicevic et al., 2023; Fagerlund et al., 2022; Fox et al., 2016). This study aims to explore the biodiversity and evolutionary characteristics of *L. monocytogenes* isolated from beef through pan-genome analysis, and to provide important reference value for its specific molecular detection. Therefore, we conducted a comparative genomics study on all *L. monocytogenes* strains isolated from beef in the NCBI database. A pan-genomic analysis and Multilocus Sequence Type (MLST) phenotypic analysis were performed for each *L. monocytogenes* strain. Pan-genomic analysis enables the identification of potential target genes in the strains. Functional analysis of the potential target genes in *L. monocytogenes* was conducted using Gene Ontology (GO) and Kyoto Encyclopedia of Genes and Genomes (KEGG) annotations. Furthermore, a protein–protein interaction (PPI) network was constructed for potential target genes of *L. monocytogenes*, and eight different hub gene analysis methods were utilized to screen novel target genes from the potential target genes. Finally, the virulence genes, antimicrobial resistance genes, CRISPR-Cas system, plasmids, and environmental resistance genes of each *L. monocytogenes* strain were investigated to explore their biodiversity and genetic determinants.

## Materials and methods

### Data availability and processing

A total of 356 genomic sequences were retrieved and downloaded from the NCBI Genomic Database (last accessed on July 13, 2024), including 344 *L. monocytogenes* strains isolated from beef, as well as genomic sequences from 5 other *Listeria* species and 7 *non-Listeria* bacterial species. The comparative genomic analysis of *L. monocytogenes* with 5 other *Listeria* species effectively distinguishes *L. monocytogenes* from other *Listeria* species. Similarly, the comparative genomic analysis with 7 *non-Listeria* bacterial species allows for effective differentiation of *L. monocytogenes* from *non-Listeria* bacteria. The concrete information of *L. monocytogenes* in research is summarized in Supplementary Tables S1, S2, including GenBank accession numbers, strain names, genome size, GC content, number of contigs and N50.

### Pan-genomics analysis of *Listeria monocytogenes* strains isolated from beef and non-targeted strains

The analysis of pan-genomic comparison of *L. monocytogenes* and non-target strains can be used to screen potential target genes. The potential target genes refer to those genes that are unique to *L. monocytogenes* strains and are absent in non-target strains (Li et al., 2021; Zhang et al., 2024). Our study found that the commonly used molecular detection target gene, *hly*, is not present in all strains of *L. monocytogenes*. Therefore, it is essential to identify potential target genes that are present in all strains of *L. monocytogenes* as new detection targets (Dapgh and Salem, 2022; Ata et al., 2017; Li et al., 2021). Perform pan-genomic analysis on 344 target strains of *L. monocytogenes* and 12 non-target reference strains. In brief, all analyzed genome sequences were annotated using Prokka v1.14.6 (Seemann, 2014), and the output results of Prokka were used for pan-genomic analysis with Roary v3.11.2 (Page et al., 2015). A core genome was determined for each isolate using a 99% cutoff, with a BLASTP identity cutoff of 85% (Pang et al., 2019). Genes that matched with all *L. monocytogenes* strains genome sequences were considered highly conserved and used for subsequent comparisons with other *Listeria* species and *non-Listeria* bacterial genomes.

Pan-genome clusters were defined as core-genes: present in all isolates; soft-core genes: present in at least 95% of isolates; shell-genes (accessory genes): present between 15 and 95% of isolates; and cloud-genes (unique genes): present in less than 15% of isolates (Mafuna et al., 2022).

The potential target genes were screened according to the following criteria: 100% presence in *L. monocytogenes* strains and no presence in non-target bacterial strains. Then, these potential target genes were used screened against the nucleotide collection (nr/nt) databases using the online BLAST program to ensure specificity (Zhang et al., 2024).

### MLST and phylogenetic analysis

Perform MLST analysis on the genome of *L. monocytogenes* to predict sequence types (STs), clonal complexes (CCs), and lineages. This analysis involved retrieving seven housekeeping genes from each *L. monocytogenes* genome using the MLST database: *abcZ* (ABC transporter), *bglA* (beta-glucosidase), *cat* (catalase), *dapE* (succinyldiaminopimelate desuccinylase), *dat* (D-alanine aminotransferase), *ldh* (lactate dehydrogenase), and *lhkA* (histidine kinase). These genes were employed to determine the STs, CCs, and lineages of *L. monocytogenes* (Wu et al., 2016; Wang et al., 2021).

To investigate the phylogenetic relationships among 344 strains of *L. monocytogenes* isolated from beef, MEGA software was utilized to construct a phylogenetic tree based on all core single-copy genes of *L. monocytogenes* (Li et al., 2021). Additionally, the ST and CC typing of each strain were annotated on the tree.

### Functional characteristics of potential target genes

In order to investigate the functional characteristics of genes present exclusively in *L. monocytogenes* strains and absent in non-target bacterial strains (potential target genes), annotation analysis was performed using GO enrichment analysis and KEGG enrichment analysis (Adnan et al., 2022), and the results were integrated.

### PPI network analysis and screening of novel target genes

In this study, the STRING database was used to establish PPI networks, and these networks were visualized using Cytoscape v3.10.2 (Lu et al., 2022). Cytoscape is a general-purpose modeling environment for integrating biomolecular interaction networks and states. The CytoHubba function in Cytoscape was used to identify hub genes (novel target genes) from the PPI network. CytoHubba ranks genes in the PPI network using eight different algorithms, including Degree, Betweenness, BottleNeck, Closeness, Edge Percolated Component (EPC), Maximum Neighborhood Component (MNC), Maximum Clique Centrality (MCC), and Stress. The top 10 genes with the highest scores in each algorithm were selected as hub genes (Zhang et al., 2024).

### Prediction of virulence factors and antimicrobial resistance genes of *Listeria monocytogenes*

Identifying the virulence and antimicrobial resistance genes of *L. monocytogenes* is crucial for understanding its genetic determinants. Therefore, we used the Virulence Factors of Pathogenic Bacteria (VFDB) database and the Comprehensive Antibiotic Resistance Database (CARD) to predict the virulence and resistance genes of *L. monocytogenes* strains (Zhu et al., 2023), and integrated the results for presentation using a heatmap.

### Prediction of CRISPR-Cas system types and plasmids of *Listeria monocytogenes*

The presence of the CRISPR-Cas system and plasmids can facilitate the evolution of *L. monocytogenes*, contributing to our understanding of its genetics. Therefore, we used CRISPRCasFinder and the PLSDB plasmid database to predict the CRISPR-Cas system and plasmids in *L. monocytogenes* strains (Di et al., 2014; Zhang et al., 2016), and integrated the results for presentation using a heatmap.

### Prediction of *Listeria* genomic Islands and stress survival Islands of *Listeria monocytogenes*

The presence of *Listeria* genomic Islands (LGIs) and survival Islands (SSIs) can enhance the resistance of *L. monocytogenes* to harsh environments, contributing to our understanding of its adaptability. Therefore, we used the BIGSdb database to predict the LGIs and SSIs in *L. monocytogenes* strains (Mafuna et al., 2021), and integrated the results for presentation using a heatmap.

### Design and validation of specific primers for *Listeria monocytogenes*

Primers for the *bglF_2* gene sequence were designed using Primer Premier 5 software (Table 1) (He et al., 2022). The primers were synthesized by Sangon Biotech Co., Ltd., Shanghai, China. PCR experiments on some of the analyzed strains to detect primer specificity. Total reaction volume was 25 μL, including 12.5 μL of 2 × Es Taq MasterMix (CWBIO, Beijing, China), 1 μL each of for ward and reverse primers (10 μM), 8.5 μL of sterile water, and 2 μL of the purified bacterial genomic DNA as a template. An equal volume of sterile distilled water was used instead of the template as a negative control. PCR thermal cycling involved an initial denaturation step at 94°C for 5 min, followed by 35 cycles of denaturation at 94°C for 30 s, annealing at 55°C for 30 s, and elongation at 72°C for 1 min, with a final elongation at 72°C for 10 min. PCR products were evaluated by 2% agarose electrophoresis.

**Table 1:** Specific target gene and primers used for the detection of *L. monocytogenes* isolated from beef.

Gene	Sequence length/bp	Primer	Sequence (5′/3′)	Encoded protein	Product size/bp
bglF_2	1,853	bglF_2F	TCGGAAATGACGTGCCTAAAGTGTT	PTS system beta-glucoside-specific EIIBCA component	464
		bglF_2R	ATCGGAATAACAGAGTAAGC		

### Artificial contamination experiments

To ensure the specificity and efficacy of the *bglF_2* gene, the primers designed for this gene were validated through artificial contamination experiments to assess their effectiveness in detecting *L. monocytogenes* in beef samples. In brief, the *L. monocytogenes* EGD-e strain was cultured overnight, and bacterial concentration was determined using the plate count method. Beef samples (10 g) obtained from a slaughterhouse in Changchun, China were minced and exposed to ultraviolet light for 30 min to ensure sterility. The samples were then added to 88 mL of Milli-Q water and incubated for 30 min. The mixture was centrifuged at 8,000 rpm for 20 min, and the supernatant was filtered through a 0.22 μm filter to obtain the simulated detection solution. Finally, 2 mL of the cultured EGD-e was added to the simulated detection solution, and the mixture was thoroughly mixed to complete the artificial contamination. After that, the mixtures were incubated at 37°C for 4 h,6 h, 8 h,12 h, and 24 h. Genomic DNA was extracted at the indicated time points from sample, and then analyzed by PCR (Li et al., 2021).

## Results

### Genome statistics and general features

In the NCBI database, we downloaded the whole-genome sequences of all *L. monocytogenes* strains isolated from beef, totaling 344 strains, and compiled the corresponding information for these strains (Supplementary Tables S1, S2). They possess an average genome size of 3.061 (2.9–3.6) Mbp, an average GC content of 37.986 (37.5–38)%, the number of contigs ≤ 312, and an average N50 of 387.121 Kbp.

### Pan-genomic analysis results

**Figure 1:**
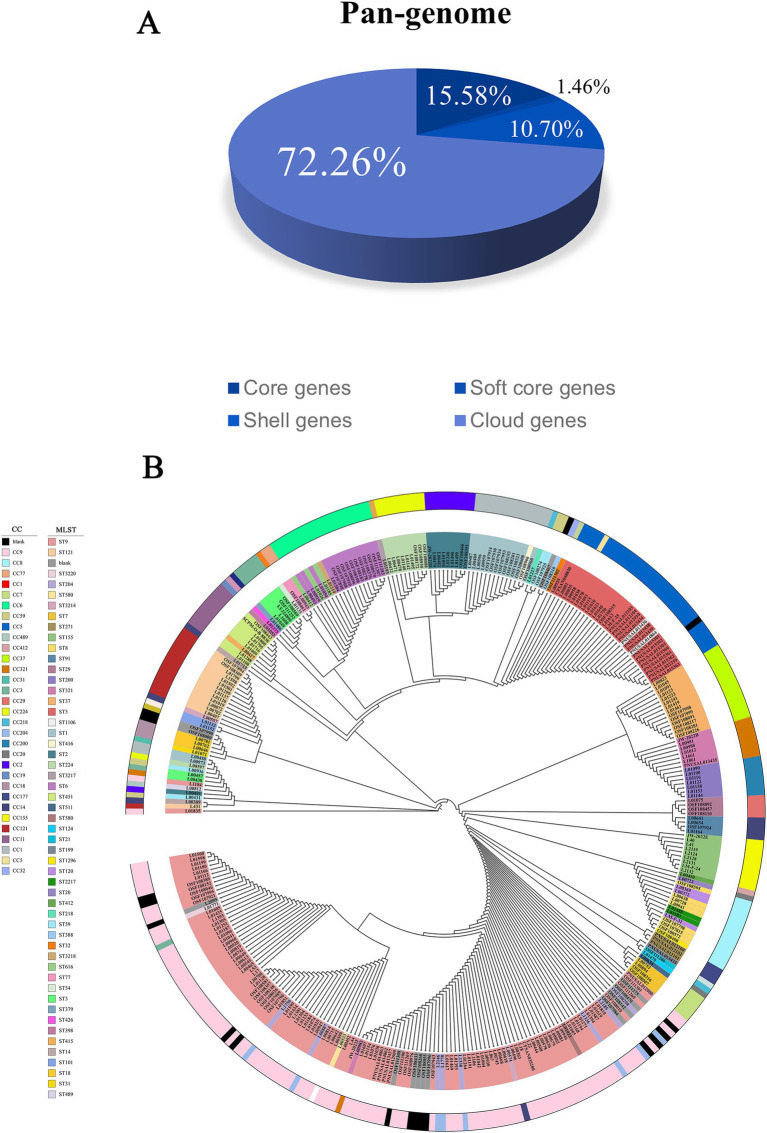
The pan-genome composition and phylogenetic evolution tree of *L. monocytogenes* isolated from beef. **(A)** The proportion of each component in the pan-genome. **(B)** The phylogenetic tree of *L. monocytogenes* isolated from beef, with the inner ring color representing different STs and the outer ring color representing different CCs.

According to the pan-genome analysis, the distribution of pan-genes in 344 *L. monocytogenes* strains is as follows: 2001 core genes (15.58%), 187 soft core genes (1.46%), 1,374 shell genes (10.70%), and 9,278 cloud genes (72.26%) (Figure 1A). As the number of strains increases, the number of pan-genes gradually increases, while the number of core genes tends to stabilize, indicating that *L. monocytogenes* possesses an open genome, which is closely related to their adaptability and virulence expression in beef environments under different conditions. To identify potential target genes, we also performed pan-genome analysis on 344 *L. monocytogenes* strains and 12 non-target strains. The results of the pan-genome analysis are as follows: the number of core genes is 0, soft core genes are 2,098, shell genes are 1,439, cloud genes are 41,690, and the total number of pan-genes is 45,227. Due to the presence of non-target strains, no common core genes were identified. However, potential target genes can be screened by comparing genes present in the non-target strains. Based on the aforementioned screening method, a total of 416 potential target genes were identified in *L. monocytogenes* strains (Supplementary Table S3). These potential target genes were present in 100% of the *L. monocytogenes* strains isolated from beef, but absent in non-target strains. These potential target genes may serve as novel target genes for *L. monocytogenes* strains isolated from beef, but further screening is required to validate these potential target genes.

### MLST and phylogenetic analysis

By uploading the whole-genome sequences of 344 *L. monocytogenes* strains to the database for comparison, these strains were classified into 52 distinct ST types and 34 different CC types. However, 18 strains did not successfully match any corresponding types in the database. The most common ST types were ST9 (28.20%) and ST5 (8.43%), while the most common CC type was CC9 (30.23%). The strains were divided into two evolutionary lineages: lineage I (30.23%) and lineage II (64.24%) (Figure 1B; Supplementary Table S4).

To elucidate the evolutionary patterns of *L. monocytogenes* strains isolated from beef, we performed a phylogenetic analysis of *L. monocytogenes* using the conserved amino acid sequences of all single-copy genes (Figure 1B). Phylogenetic tree analysis revealed that the *L. monocytogenes* strains isolated from beef share a common ancestor and belong to the main root of the *Listeria* genus. Most *L. monocytogenes* strains isolated from the same region clustered into the same branch, suggesting a distinct regional pattern in the evolution of *L. monocytogenes*. However, some strains, although isolated from different regions, were grouped into the same branch, indicating a certain level of evolutionary similarity and strong adaptability of *L. monocytogenes* to different environments.

### Enrichment analysis of the functional characteristics of potential target genes using the GO and KEGG databases

**Figure 2:**
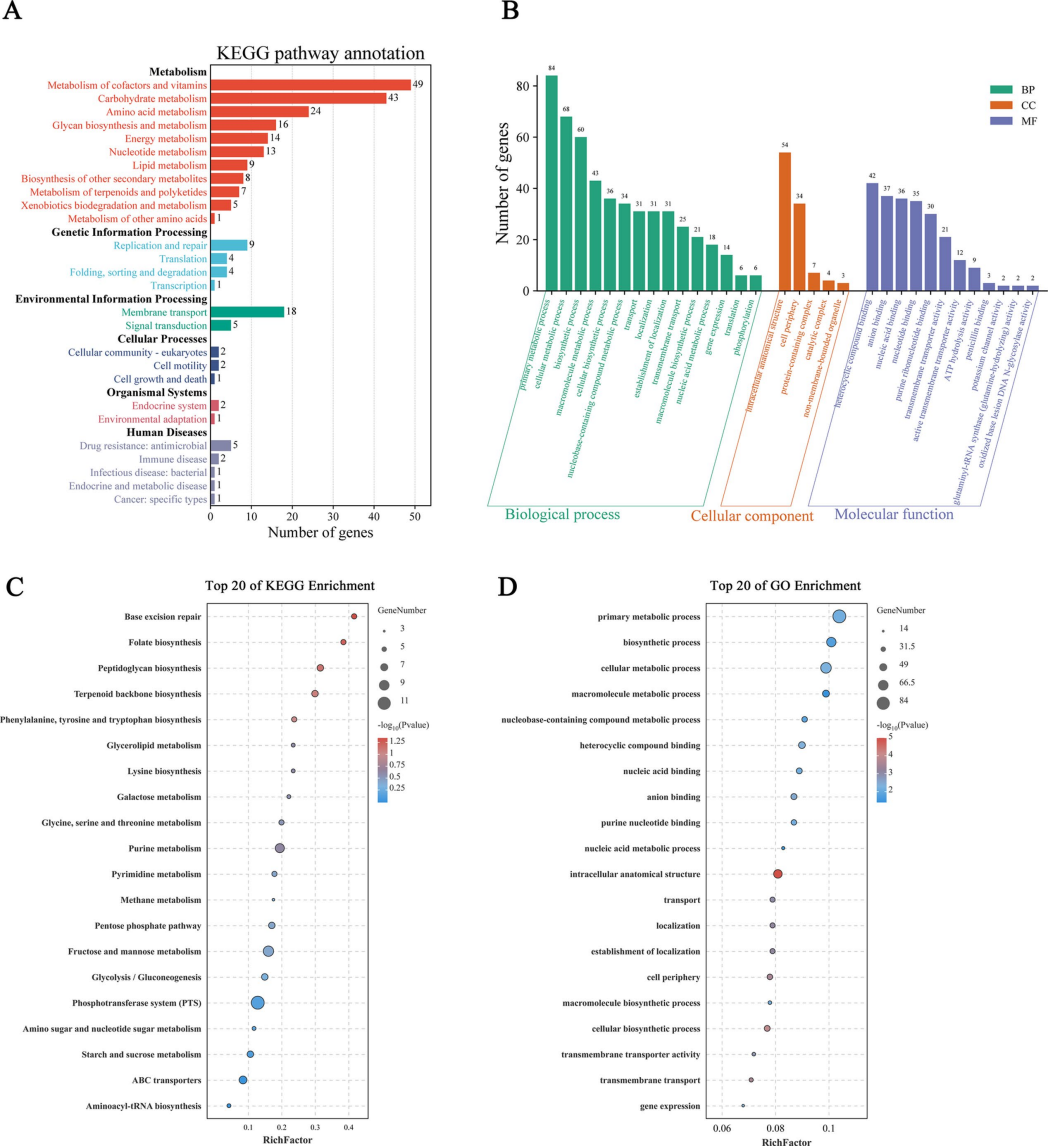
Enrichment analysis of potential target genes of *L. monocytogenes* isolated from beef based on GO and KEGG databases. **(A)** Enrichment analysis based on the KEGG database. **(B)** Enrichment analysis based on the GO database. **(C)** Enrichment analysis based on KEGG database with the top 20 enriched terms listed. **(D)** Enrichment analysis based on GO database with the top 20 enriched terms listed.

To investigate the functional characteristics of the 416 potential target genes from *L. monocytogenes* strains isolated from beef, we performed functional annotation and classification of these genes using the GO and KEGG databases. The detailed information of the potential target genes is presented in Supplementary Table S3. The pathway database of KEGG is the most widely used public database for metabolic pathways, which classifies biological metabolic pathways into six categories: Metabolism, Genetic Information Processing, Environmental Information Processing, Cellular Processes, Organismal Systems, and Human Diseases. The potential target genes were annotated according to six pathway categories in the KEGG database. Among these, the most enriched pathways in the Metabolism category were metabolism of cofactors and vitamins (*n* = 49, 11.78%), carbohydrate metabolism (*n* = 43, 15.87%), and amino acid metabolism (*n* = 24, 5.78%). In the Genetic Information Processing category, replication and repair (*n* = 9, 2.16%) was the most enriched pathways. In the Environmental Information Processing category, membrane transport (*n* = 18, 4.33%) and signal transduction (*n* = 5, 1.20%) were the most enriched pathways. In the Cellular Processes category, cellular community—eukaryotes (*n* = 2, 0.48%) and cell motility (*n* = 2, 0.48%) were the most enriched pathways. In the Organismal Systems category, endocrine system (*n* = 2, 0.48%) was the most enriched pathways. In the Human Diseases category, drug resistance: antimicrobial (*n* = 5, 1.2%) was the most enriched pathways (Figure 2A). We integrated and ranked the KEGG enrichment analysis results of all potential target genes, and generated bubble maps to show the top 20 functional features in KEGG enrichment analysis based on the significance of gene number and *p*-value (Figure 2C).

The GO database categorizes gene functions into three main categories, namely Biological Processes (BP), Cellular Components (CC), and Molecular Functions (MF). In the BP category, the most enriched biological processes were primary metabolic process (*n* = 84, 20.19%), cellular metabolic process (*n* = 68, 16.35%), biosynthetic process (*n* = 60, 14.42%), and macromolecule metabolic process (*n* = 43, 10.34%). Within the CC category, the most abundant cellular components were intracellular anatomical structure (*n* = 54, 12.98%), cell periphery (*n* = 34, 8.17%). In the MF category, the most enriched molecular functions were heterocyclic compound binding (*n* = 42, 10.09%), and anion binding (*n* = 37, 8.89%) (Figure 2B). We integrated and ranked all the GO enrichment analysis results of potential target genes, and generated a bubble chart to display the top 20 functional features in GO enrichment analysis based on the number of genes and the significance of *p*-values (Figure 2D).

Enrichment analysis of the functional characteristics of the 416 potential target genes using the GO and KEGG databases revealed that these genes are primarily associated with metabolic processes, compound binding, protein localization, and transmembrane transport in *L. monocytogenes*. Specifically, these genes are involved in cellular metabolic processes, carbohydrate metabolism, and biosynthesis of organic matter. For example, carbohydrate metabolism is critical for energy acquisition in *L. monocytogenes* because it involves converting sugars into a form of energy that cells can use directly. These potential target genes are closely related to the basic biological processes of *L. monocytogenes* and the pathogenesis of infection. They not only support the basic metabolic activities of the bacteria, but also involve the invasion and pathogenic effects of the bacteria on the host, including how the bacteria survive in the host cell, replicate and evade the innate immune response of the host. However, there were still some genes with unclear functional information, which warrants further investigation in future studies. In conclusion, the functional characteristics of these genes provide deeper insights into the biological properties of *L. monocytogenes* and offer potential molecular targets for the development of new control strategies.

### PPI network analysis of potential target genes and identification of novel target genes

**Figure 3:**
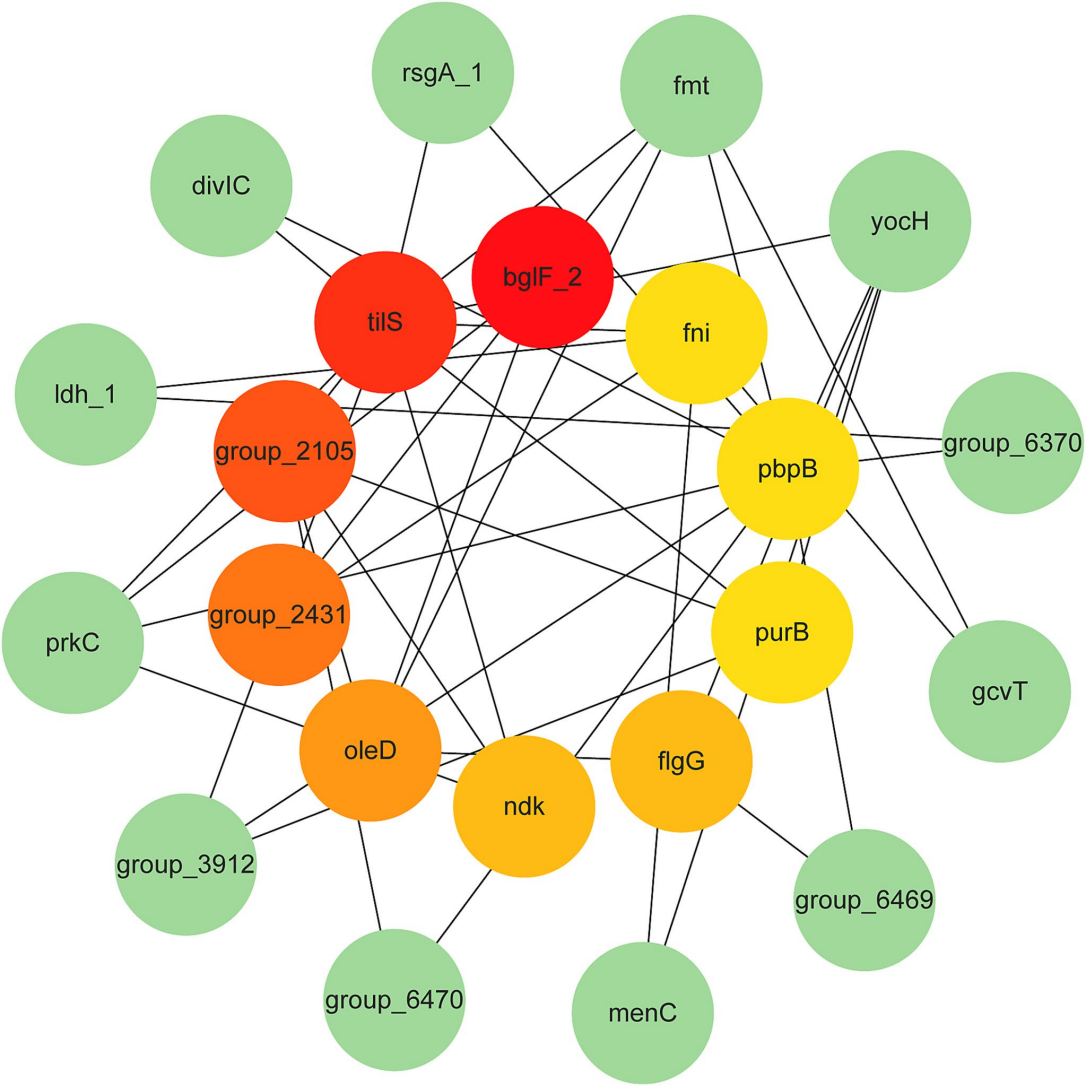
PPI network analysis of novel target genes of *L. monocytogenes* isolates from beef. The darker the red, the higher the gene score.

To further explore the relationship between potential target genes of *L. monocytogenes* PPI analysis was carried out using the STRING database. The PPI network of potential target genes comprised 416 genes, and visualization of the PPI network was performed using Cytoscape v3.10.1 software. Cytoscape is a general-purpose modeling environment for integrating biomolecular interaction networks and states. Potential target genes in the PPI network were screened using eight different algorithms in the CytoHubba plugin of Cytoscape software. The top 10 genes with the highest scores were selected as hub genes, and their rankings are shown in Table 2. Finally, the top 10 hub genes with the highest scores obtained from the Degree algorithm were identified as novel target genes. A PPI network was constructed based on their scores. These 10 hub genes include *bglF_2*, *tilS*, *group_2105*, *group_2431*, *oleD*, *ndk*, *flgG*, *purB*, *pbpB*, and *fni* (Figure 3). Detailed information on these 10 hub genes, including their functional roles, gene lengths, and other characteristics, is presented in Table 3. These 10 hub genes play a crucial role in maintaining the basic life activities and infection processes of *L. monocytogenes* strains and may serve as novel target genes for *L. monocytogenes*, particularly the top-scoring gene, *bglF_2*.

**Table 2:** Top 10 hub genes ranked by scoring in eight different algorithms.

Catelogy	Rank methods in cytoHubba							
	Betweenness	BottleNeck	Closeness	Degree	EPC	MCC	MNC	Stress
Gene symbol top 10	group_2105	group_2105	tilS	bglF_2	bglF_2	gatY_3	bglF_2	tilS
	tilS	ndk	group_2105	tilS	tilS	gatY_2	tilS	group_2105
	bglF_2	flgG	bglF_2	group_2105	oleD	frwD	group_2431	bglF_2
	flgG	bglF_2	oleD	group_2431	group_2431	mngA_3	gatY_3	ndk
	ndk	group_5668	ndk	oleD	purB	bglF_2	gatY_2	flgG
	oleD	tilS	group_2431	ndk	ndk	yhaP	cpoA	oleD
	group_2431	oleD	prkC	flgG	group_2105	mshD_2	purB	group_2431
	group_5795	fmt	pyrE	purB	pyrE	group_10780	group_11281	cysG
	prkC	hemH	fmt	pbpB	gatY_3	pgcA_1	group_5806	pphA
	cysG	cysG	purB	fni	pgcA_1	pgcA_2	group_45051	group_5795

bglF_2 gene highlighted in bold are the most promising candidate to serve as novel target genes.

**Table 3:** Detailed information of top 10 hub genes ranked by scoring in Degree algorithm.

Gene	Name of target ganes	Sequence length/bp	Presence profile		Encoded protein	Source
			In target	In non-target		
bglF_2	lmo2772	1,853	344 (100%)	0	PTS system beta-glucoside-specific EIIBCA component	This study
tilS	lmo0219	1,945	344 (100%)	0	tRNA(Ile)-lysidine synthase	This study
group_2105	lmo1266	955	344 (100%)	0	O-succinylbenzoic acid--CoA ligase	This study
group_2431	lmo0078	1,350	344 (100%)	0	Endonuclease MutS2	This study
oleD	lmo1477	1,027	344 (100%)	0	Putative N-acetyl-LL-diaminopimelate aminotransferase	This study
ndk	ndk	443	344 (100%)	0	5-dehydro-2-deoxygluconokinase	This study
flgG	lmo0682	779	344 (100%)	0	Glutathione biosynthesis bifunctional protein GshAB	This study
purB	purB	1,292	344 (100%)	0	Siroheme synthase	This study
pbpB	pbpB	2,216	344 (100%)	0	Tryptophan synthase alpha chain	This study
fni	lmo1383	1,073	344 (100%)	0	Histidinol-phosphate aminotransferase	This study

### Prediction results of virulence and antimicrobial resistance genes

**Figure 4:**
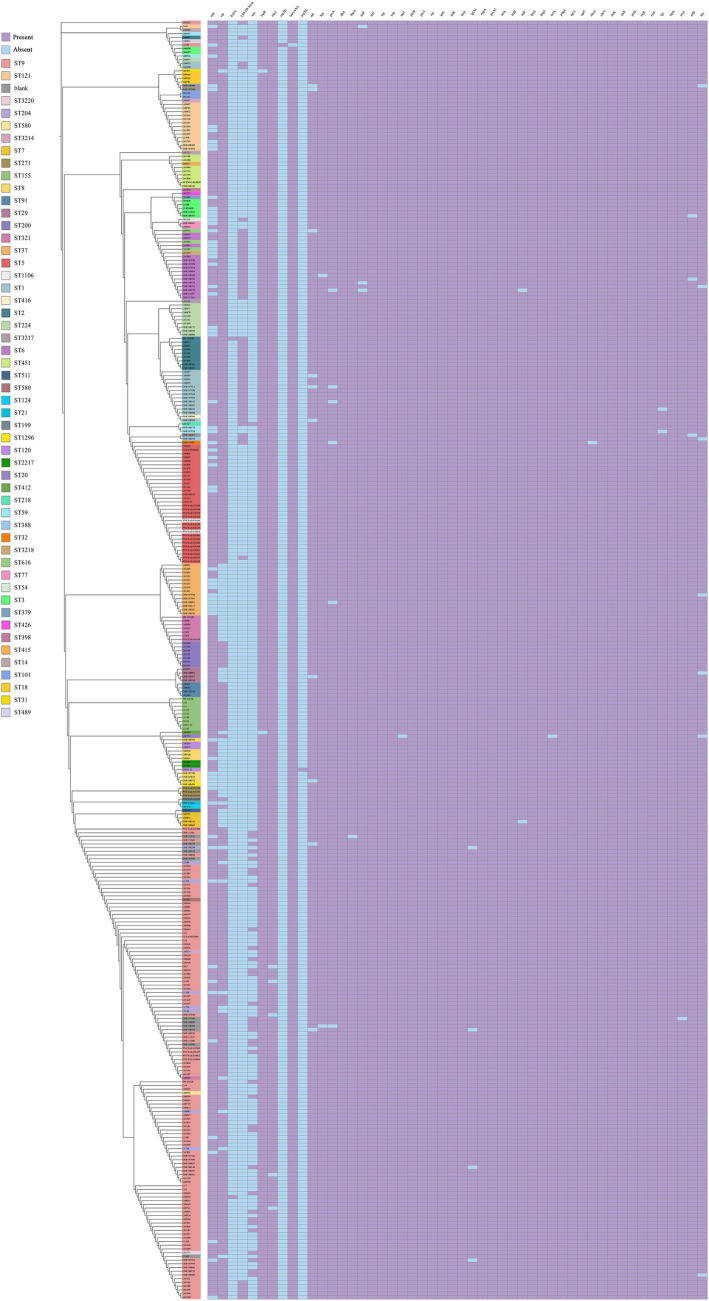
The distribution of virulence genes in *L. monocytogenes* isolated from beef.

To investigate the virulence relationships and pathogenic mechanisms among *L. monocytogenes* strains isolated from beef, we predicted the virulence factor encoding genes in the whole genome of *L. monocytogenes* strains. Based on VFDB prediction and annotation, virulence factors of *L. monocytogenes* were classified into 12 categories including Adherence, Bile resistance, Enzyme, Immune modulator, Intracellular survival, Invasion, Iron uptake, Nucleation-promoting factor, Peptidoglycan modification, Regulation, Surface protein anchoring, and Toxin. The VFDB prediction revealed that 26 virulence genes were present in all *L. monocytogenes* strains isolated from beef, including *dltA*, *inlJ*, *lap*, *bsh*, *plcB*, *plcA*, *stp*, *inlC*, *inlK*, *lntA*, *oppA*, *prsA2*, *inlA*, *inlB*, *lpeA*, *hbp2*, *pdgA*, *agrA*, *agrC*, *cheY*, *lisK*, *lisR*, *prfA*, *virR*, *virS*, and *lspA* (Figure 4). During the process of invading and infecting the host, the genes *inlA*, *inlB*, *plcA*, *plcB, prfA*, *hly*, and *actA* play critical roles. In this study, the *inlA*, *inlB*, *plcA*, *plcB*, and *prfA* genes were present in 100% of the *L. monocytogenes* strains, while the *hly* and *actA* genes were not present in all strains, but their presence probability exceeded 97%. The prediction of virulence genes indicates that *L. monocytogenes* strains isolated from beef contain a large number of virulence genes, suggesting that these strains possess high virulence and pathogenicity.

**Figure 5:**
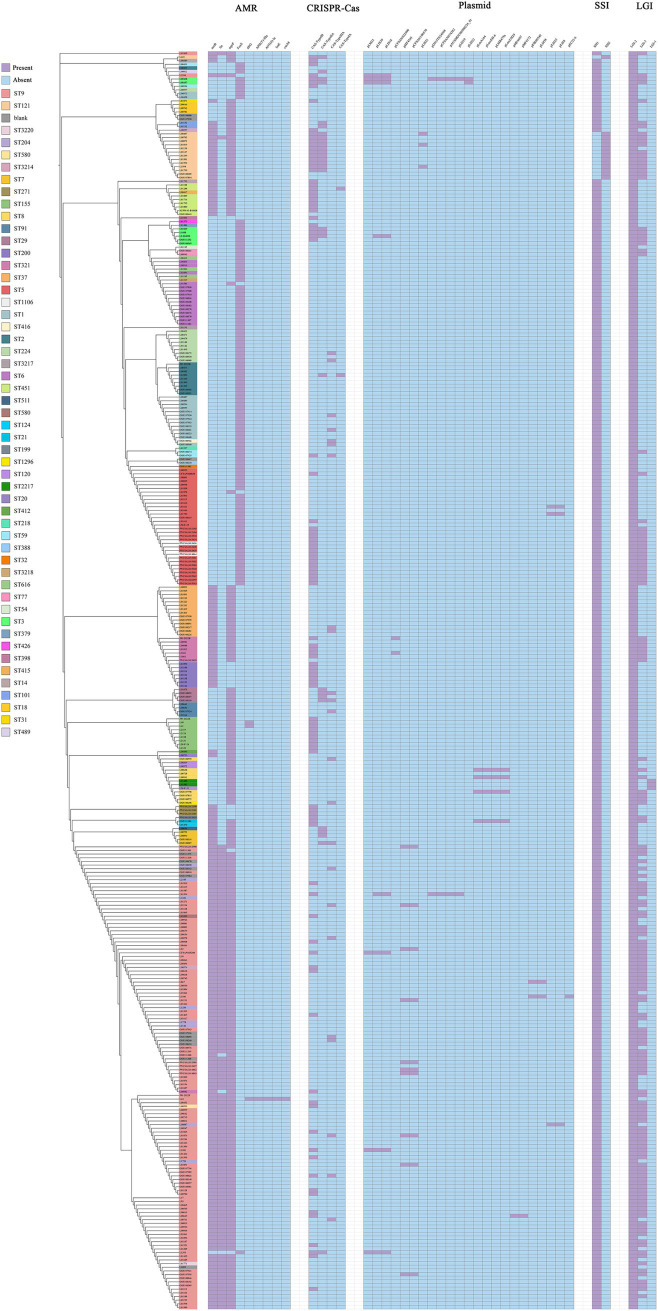
The distribution of antimicrobial resistance genes, CRISPR-Cas system, plasmids, SSIs and LGIs in *L. monocytogenes* isolated from beef.

With the widespread use of antibiotics, bacterial resistance has become a major concern. The changes in antimicrobial resistance genes during bacterial evolution help us gain a deeper understanding of the trends in bacterial antimicrobial resistance. Using the CARD database, a total of 9 antimicrobial resistance genes were predicted in 344 *L. monocytogenes* strains isolated from beef, belonging to 9 drug categories and exhibiting 5 resistance mechanisms. The identified antimicrobial resistance genes include *norB*, *lin*, *mprF*, *FosX*, *dfrG*, *APH(3′)-IIIa*, *ANT(6)-Ia*, *lsaE*, and *catA8* (Figure 5). The most common 4 antimicrobial resistance genes in *L. monocytogenes* strains isolated from beef are *norB*, *lin*, *mprF*, and *FosX*, indicating high resistance to fluoroquinolones, lincosamides, peptides and phosphonic acid antibiotics.

### Prediction results of CRISPR-Cas system types and plasmids

The CRISPR-Cas system is an adaptive immune system in bacteria that defends against the invasion of foreign genetic material, such as phages and plasmids. It serves as a defense mechanism found in most bacteria to eliminate foreign plasmid or phage DNA. An analysis of the CRISPR-Cas system in 344 *L. monocytogenes* strains isolated from beef identified 4 types of CRISPR-Cas systems, each exhibiting distinct Cas genes. The CRISPR-Cas system types detected in *L. monocytogenes* included CAS-TypeIA (2/344), CAS-TypeIB (105/344), CAS-TypeIIA (30/344), and CAS-TypeIIIA (23/344). In total, 39.24% (135/344) of the *L. monocytogenes* strains contained at least one CRISPR-Cas system, with CAS-TypeIB (30.52%) and CAS-TypeIIA (8.72%) being the most prevalent (Figure 5). CAS-TypeIA was detected only in ST451 and ST2.

Mobile Genetic Elements (MGEs) refer to a class of genetic elements capable of spreading or transferring within a genome, such as plasmids, which can facilitate the evolution of microorganisms. Therefore, the prediction of plasmids can provide further insights into the evolution of *L. monocytogenes*. We employed PLSDB databases for the detection of plasmids, and the results only documented plasmids with an identity score of 1. A total of 23 plasmids were predicted in *L. monocytogenes* strains, with the most prevalent plasmids being pMF4545 (10/344) and pCFSAN100570 (10/344), followed by pLM33 (8/344) and pLIS18 (8/344). The plasmids pMF4545 and pCFSAN100570 were exclusively found in ST9 (Figure 5).

### Prediction results of LGIs and SSIs

We predicted the presence of genomic LGIs in *L. monocytogenes* strains and found that LGI-2 was present in all strains. LGI-1 was less common, detected in only 3 *L. monocytogenes* strains, while LGI-3 was found in 155 strains (Figure 5).

The SSIs associated with resistance genes in *Listeria* consist of 2 islands: SSI-1 and SSI-2. SSI-1 consists of the genes *lmo0444*, *lmo0445*, *pva*, *gadD*, and *gadT*, while SSI-2 is composed of the genes *lin0464* and *lin0465*. SSI-1 is associated with the tolerance of *Listeria* to environmental factors such as high NaCl concentrations, high bile salt concentrations, and low pH. In contrast, SSI-2 contributes to the survival of strains under alkaline and oxidative stress, enabling them to persist in food processing environments. Initially, the two genes of SSI-2 were thought to be unique to *L. innocua*, however, studies have found that these genes are also present in *L. monocytogenes* of the ST121. Prediction of SSIs revealed that 330 strains (95.93%) of *L. monocytogenes* harbor SSI-1, while 14 strains (4.07%) of *L. monocytogenes* harbor SSI-2 (Figure 5). It is noteworthy that all 14 *L. monocytogenes* strains harboring SSI-2 were identified as ST121. Among these 14 *L. monocytogenes* strains, 12 were isolated from the Netherlands, 1 from Spain, and 1 from Australia.

### Detection of *Listeria monocytogenes* using specific primers by PCR

**Figure 6:**
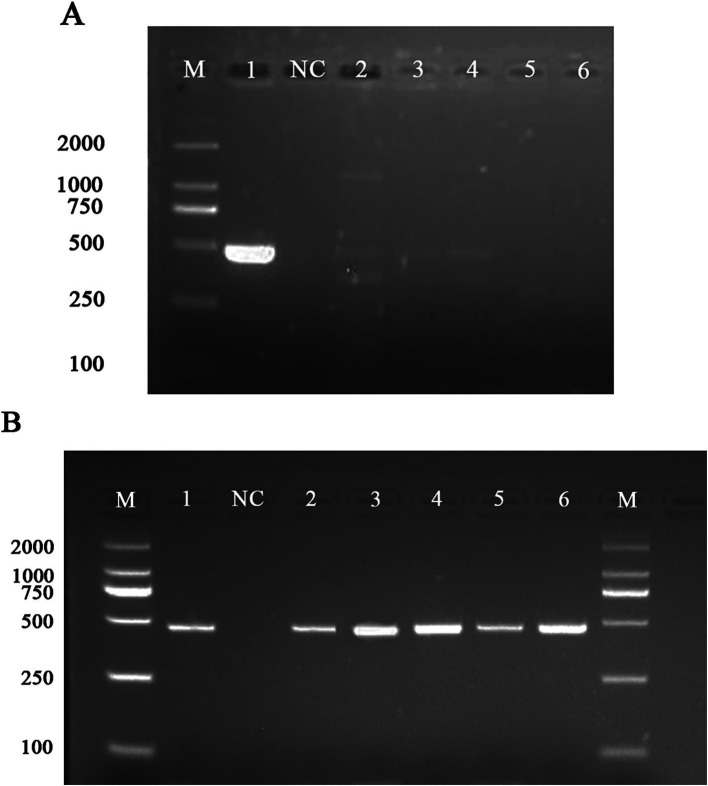
Verification of primer specificity for the *bglF_2* gene of *L. monocytogenes* and artificial contamination experiments. **(A)** Lane M: DL DNA 2000 marker, lane NC: negative control, and lanes 1–6: represent 6 different strains, including lane 1: *Listeria monocytogenes*, lane 2: *Listeria innocua*, lane 3: *Listeria ivanovii*, lane 4: *Listeria welshimeri*, lane 5: *Escherichia coli*, lane 6: *Salmonella enterica*. **(B)** Lane M: DL DNA 2000 marker, lane 1: positive control, lane NC: negative control, lane 2: DNA extracted from samples at 4 h, lane 3: DNA extracted from samples at 6 h, lane 4: DNA extracted from samples at 8 h, lane 5: DNA extracted from samples at 12 h, lane 6: DNA extracted from samples at 24 h.

To validate the specificity of the *bglF_2* gene for *L. monocytogenes*, primers targeting the *bglF_2* gene were designed, and its specificity was evaluated through PCR. PCR results showed that in the primer system designed for the *bglF_2* gene, a distinct band was observed at 464 bp only in *L. monocytogenes*, while no bands were detected in other *non-L. monocytogenes* strains (Figure 6A). The results indicate that the *bglF_2* gene exhibits good specificity for *L. monocytogenes*, and primers designed for the *bglF_2* gene can effectively detect *L. monocytogenes*.

### The results of the artificial contamination experiment

To ensure the primers designed for the *bglF_2* gene can effectively detect *L. monocytogenes* in beef samples, their specificity and efficacy were validated through artificial contamination experiments. *L. monocytogenes* at a concentration of 5.2 × 10^4^ CFU/mL was added to the simulated detection solution and subjected to 24 h of enrichment culture. DNA was extracted from the samples at 4 h, 6 h, 8 h, 12 h, and 24 h, and PCR validation was performed using primers designed for the *bglF_2* gene. The results showed distinct bands at 4 h, 6 h, 8 h, 12 h, and 24 h, indicating that the primers designed for the *bglF_2* gene can effectively detect *L. monocytogenes* in beef samples (Figure 6B). The *bglF_2* gene demonstrated excellent specificity and holds promise as a specific molecular target for detecting *L. monocytogenes* strains in beef samples.

## Discussion

Food safety is a critical issue in the field of public health, and *L. monocytogenes*, as a significant foodborne pathogen, often poses serious food safety risks (Chowdhury and Anand, 2023). Therefore, we conducted a comparative genomic analysis of *L. monocytogenes* strains isolated from beef to explore their phenotypic and genetic evaluations, revealing their biodiversity and evolutionary traits. We also identified potential target genes and mining novel targets. The aim was to gain a deeper understanding of *L. monocytogenes* strains and provide important reference value for strain-specific molecular detection, thereby reducing food safety issues in public health.

In this study, we conducted a pan-genome comparative analysis of 344 *L. monocytogenes* strains isolated from beef to investigate strain phenotypes and genetic evaluations, revealing their biodiversity and evolutionary features. To assess the genomic diversity of *L. monocytogenes* strains isolated from beef, we performed core/pan-genome analysis. The pan-genome represents all the genes present in the strains, while the core genome represents the essential portion necessary for the presence and shared phenotypic features of specific strains, which are critical for maintaining basic survival and infection capability (Lee et al., 2019). Therefore, we conducted an in-depth study of the core genome to identify potential target genes. A total of 2,001 core genes, accounting for 15.58%, were identified among all *L. monocytogenes* strains isolated from beef in the NCBI database. These genes constitute the fundamental components essential for the survival and development of *L. monocytogenes* strains. This suggests that despite evolving in different regions and environments, *L. monocytogenes* strains still share a significant number of common phenotypic features. In addition to the core genes, the pan-genome also contains many accessory genes, which provide the strains with unique traits (Nwaiwu, 2022). As *L. monocytogenes* strains are found in various environments, they have evolved specific accessory genes to adapt to these distinct environments and counteract environmental pressures. Through pangenomic analysis, we identified the core genes of *L. monocytogenes* strains isolated from beef, and subsequently used these core genes to determine potential target genes. The selection criterion for potential target genes was that they were present exclusively in *L. monocytogenes* strains isolated from beef, and absent in non-target strains. This suggests that the potential target genes are essential and unique to *L. monocytogenes*, playing a crucial role in its basic survival and pathogenic invasion. Therefore, studying the potential target genes of *L. monocytogenes* can enhance our understanding of its phenotypic and genetic evaluations, and these potential target genes could also serve as specific molecular detection targets for *L. monocytogenes*.

To investigate the biodiversity and evolutionary characteristics of *L. monocytogenes* strains isolated from beef, we performed MLST and phylogenetic analyses. MLST analysis is a crucial tool in the classification and epidemiological study of *L. monocytogenes*, providing important insights into the genetic diversity and transmission routes of *L. monocytogenes* strains (Stessl et al., 2014). Our MLST analysis of *L. monocytogenes* strains isolated from beef revealed that ST9 and CC9 were the most prevalent, followed by ST5 and CC5. Similar findings were reported by Henri et al. (2016) who conducted MLST analysis on *L. monocytogenes* isolated from food, with ST9 and ST121 being the most common. Manqele et al. (2024) performed MLST analysis of *L. monocytogenes* in beef and beef products and identified ST9, ST204, ST1, and ST5. Our results are consistent with the findings reported in the above-mentioned literature. This indicates that *L. monocytogenes* strains in beef predominantly belong to ST9 and CC9 types. By constructing a phylogenetic tree, we found that although the *L. monocytogenes* strains studied were isolated from different regions and environments, some of the strains were still grouped into the same branch. This suggests that while *L. monocytogenes* strains have evolved unique traits to adapt to the specific environmental pressures they encounter, they still exhibit a certain degree of genetic similarity in their evolutionary characteristics.

We obtained the core genes of *L. monocytogenes* strains isolated from beef through pan-genomic analysis, and then identified 416 potential target genes by screening these core genes. To explore the functional roles of these potential target genes, we conducted GO and KEGG enrichment analyses (Zhang et al., 2024). The functional annotation results indicated that the potential target genes are mainly associated with the metabolic processes, compound binding, protein localization, and transmembrane transport of *L. monocytogenes*, which are closely related to the bacterium’s basic life activities and its ability to invade and infect hosts. The potential target genes are promising candidates for specific targeting of *L. monocytogenes* (Zhang et al., 2024). However, due to the large number of potential target genes, further screening was performed. A PPI network was constructed, and the hub genes were identified using the Cytohubba function in Cytoscape software. Hub genes are the most critical genes in the PPI network and are used to discover novel target genes (Zhang et al., 2024). Ultimately, 10 hub genes were selected from the potential target genes (*bglF_2*, *tilS*, *group_2105*, *group_2431*, *oleD*, *ndk*, *flgG*, *purB*, *pbpB*, and *fni*). These hub genes play a crucial role in the basic life activities and infection invasiveness of *L. monocytogenes*. Among them, the *bglF_2* gene scored the highest and exhibited stronger connections with other proteins, indicating its potential as a specific molecular target for *L. monocytogenes* detection.

*L. monocytogenes* is a recognized pathogenic strain of *Listeria* (Disson et al., 2021). To further understand the genetic determinants behind the virulence of *L. monocytogenes*, we predicted the virulence genes of *L. monocytogenes* strains isolated from beef. During invasion of the host by *L. monocytogenes*, the bacterium first utilizes the *inlA* and *inlB* genes to bind with the E-Cadherin and Met receptors of the host’s eukaryotic cell membrane, respectively, thereby inducing bacterial uptake through receptor-mediated endocytosis. After internalization, the bacterium is encapsulated within a vacuole, and releases the *hly*, *plcA*, and *plcB* genes to mediate vacuole escape. Subsequently, the *actA* gene is utilized to induce actin polymerization and generate sufficient force for the bacterium to spread from one cell to another (Osek and Wieczorek, 2022). Our predictive results revealed that 50 virulence genes are present in *L. monocytogenes* strains isolated from beef, with 26 of these virulence genes being consistently present in all strains. These include key virulence genes involved in the infection and invasion process of *L. monocytogenes*, such as *inlA*, *inlB*, *plcA*, *plcB*, and *prfA* genes. Interestingly, *hly* and *actA* genes were not present in all strains, although their occurrence exceeded 97%. This discrepancy could be attributed to prediction errors in the database or genetic variations occurring in individual strains under specific environmental conditions. The presence of a large number of virulence genes contributes to the strong pathogenicity of *L. monocytogenes*, making it a virulent strain. The genetic determinants behind *L. monocytogenes* virulence are governed by its virulence genes. These results suggest that although some virulence genes may undergo genetic variation during the evolutionary process, critical virulence genes essential for the infection and invasion of the host remain conserved, playing a crucial role in *L. monocytogenes* pathogenicity.

To further investigate the genetic determinants behind the antibiotic and environmental resistance of *L. monocytogenes*, we predicted the presence of antimicrobial resistance genes, CRISPR-Cas systems, plasmids, SSIs, and LGIs in *L. monocytogenes* strains isolated from beef. Antibiotic resistance has long been a concerning issue (Asghar et al., 2024). Our predictions revealed that the most common antibiotic resistance genes in *L. monocytogenes* are *norB*, *lin*, *mprF*, and *FosX*, indicating high resistance to fluoroquinolones, lincosamides, peptides, and phosphonic acid antibiotics. The recommended antibiotics for treating *L. monocytogenes* infections are ampicillin and gentamicin. The CRISPR-Cas system is an important adaptive immune mechanism in bacteria, used to defend against foreign genetic elements such as phages and plasmids (Liu et al., 2024). Our predictions revealed the presence of a significant number of CAS-TypeIB and CAS-TypeIIA in *L. monocytogenes* strains, suggesting that *L. monocytogenes* evolves an environment-specific CRISPR-Cas system during its evolutionary process. Predicting the presence of plasmids in *L. monocytogenes* helps analyze the variations in MGEs and provides further insights into the evolution of the strains. Our prediction of plasmids revealed that, despite *L. monocytogenes* being exposed to different external environments, there remains a certain degree of similarity in bacterial genome evolution. The prediction of SSI and LGI in *L. monocytogenes* provides valuable insights into the changes in the environmental resistance of the bacterium (Hein et al., 2011). We found that LGI-2 was present in all strains, whereas LGI-3 was only present in 155 strains, suggesting that the presence of LGI-2 plays a crucial role in the resistance to environmental stress during the evolution of *L. monocytogenes*. SSI prediction revealed that SSI-1 was present in the majority of *L. monocytogenes* strains, whereas SSI-2 was found only in the ST121 strains, indicating that the presence of SSI-1 helps *L. monocytogenes* survive in harsh environments. Therefore, LGI-2 and SSI-1 are a genetic determinant for environmental resistance in the evolution of *L. monocytogenes*.

To validate the specificity of the *bglF_2* gene, primers were designed for the *bglF_2* gene, and its specificity was confirmed through PCR. The results indicate that the *bglF_2* gene exhibits excellent specificity for *L. monocytogenes*. The rationale for selecting genes different from those used in previous studies for detecting *L. monocytogenes* is that the *bglF_2* gene yielded the best results in the analysis of *L. monocytogenes* strains isolated from beef (Zhang et al., 2024). Meanwhile, the *bglF_2* gene, as a component of the PTS system, is typically strain-specific, which helps to avoid cross-reactivity with other *Listeria* species and *non-Listeria* bacteria. Therefore, the *bglF_2* gene exhibits better specificity in these strains, making it a more suitable choice for detecting *L. monocytogenes* in beef. Subsequently, an artificial contamination experiment was conducted to verify whether the *bglF_2* gene could successfully detect *L. monocytogenes* in beef samples. The results showed that the *bglF_2* gene effectively detected *L. monocytogenes* at five different time points, indicating that *bglF_2* not only efficiently detects *L. monocytogenes* in beef samples but also performs excellently. Therefore, the *bglF_2* gene is expected to be a specific molecular target for detecting *L. monocytogenes* in beef samples.

## Conclusion

In conclusion, we conducted a comparative genomics study to explore the phenotypic and genetic evaluations of *L. monocytogenes* strains isolated from beef, revealing the biodiversity and evolutionary traits of these strains. A large number of virulence genes were identified in the *L. monocytogenes* strains, which form the basis for the high pathogenicity of *L. monocytogenes*. Although the strains analyzed are from different environments and have evolved unique genes to cope with various environmental pressures, they all isolated from beef, and thus share many common features in their evolutionary process. These common features allowed us to identify potential target genes, which were further explored to discover novel targets. Specificity tests and artificial contamination experiments confirmed that the *bglF_2* gene holds promise as a specific molecular target for detecting *L. monocytogenes* strains in beef samples. This study further enhances our understanding of the pathogenicity and adaptability of *L. monocytogenes*, while providing significant reference value for the development of specific molecular detection targets for this pathogen.

## Data Availability

The original contributions presented in the study are included in the article/Supplementary material, further inquiries can be directed to the corresponding author.

## References

[ref1] Adnan M. Siddiqui A. J. Noumi E. Hannachi S. Ashraf S. A. Awadelkareem A. M.. (2022). Integrating network pharmacology approaches to decipher the multi-target pharmacological mechanism of microbial biosurfactants as novel green antimicrobials against Listeriosis. Antibiotics (Basel) 12:5. doi: 10.3390/antibiotics12010005, PMID: 36671206 PMC9854906

[ref2] Asghar A. Khalid A. Baqar Z. Hussain N. Saleem M. Z. Sairash. (2024). An insights into emerging trends to control the threats of antimicrobial resistance (AMR): an address to public health risks. Arch. Microbiol. 206:72. doi: 10.1007/s00203-023-03800-9, PMID: 38252323

[ref3] Ata N. S. Abuelnaga A. S. M. Atta N. S. Hedia R. M. Elgabry E. A. Ibrahim E. S.. (2017). Existence and virulence designation of *Listeria Monocytogenes* in retail chilled pork byproducts in Cairo porcine markets with trials of using Lactobacillus probiotic as anti-Listerial meat Perservative. IOSR J. Environ. Sci. Toxicol. Food Technol. 11, 19–23. doi: 10.9790/2402-1105041923

[ref4] Buszewski B. Rogowska A. Pomastowski P. Złoch M. Railean-Plugaru V. (2017). Identification of microorganisms by modern analytical techniques. J. AOAC Int. 100, 1607–1623. doi: 10.5740/jaoacint.17-0207, PMID: 28703095

[ref5] Chowdhury B. Anand S. (2023). Environmental persistence of *Listeria monocytogenes* and its implications in dairy processing plants. Compr. Rev. Food Sci. Food Saf. 22, 4573–4599. doi: 10.1111/1541-4337.13234, PMID: 37680027

[ref6] Colomba C. Rubino R. Anastasia A. Palermo G. Lo P. D. Abbott M.. (2020). Postpartum listeria meningitis. IDCases 21:e00896. doi: 10.1016/j.idcr.2020.e00896, PMID: 32670794 PMC7347977

[ref7] Dapgh A. Salem R. L. (2022). Molecular detection of *Listeria monocytogenes* in Milk and some Milk products. Int. J. Vet. Sci. 11, 514–519. doi: 10.47278/journal.ijvs/2021.128

[ref8] Di H. Ye L. Yan H. Meng H. Yamasak S. Shi L. (2014). Comparative analysis of CRISPR loci in different *Listeria monocytogenes* lineages. Biochem. Biophys. Res. Commun. 454, 399–403. doi: 10.1016/j.bbrc.2014.10.018, PMID: 25445602

[ref9] Disson O. Moura A. Lecuit M. (2021). Making sense of the biodiversity and virulence of *Listeria monocytogenes*. Trends Microbiol. 29, 811–822. doi: 10.1016/j.tim.2021.01.008 33583696

[ref10] Fagerlund A. Idland L. Heir E. Møretrø T. Aspholm M. Lindbäck T.. (2022). Whole-genome sequencing analysis of *Listeria monocytogenes* from rural, urban, and farm environments in Norway: genetic diversity, persistence, and relation to clinical and food isolates. Appl. Environ. Microbiol. 88:e0213621. doi: 10.1128/aem.02136-21, PMID: 35108102 PMC8939345

[ref11] Feng Y. Yao H. Chen S. Sun X. Yin Y. Jiao X. (2020). Rapid detection of Hypervirulent Serovar 4h *Listeria monocytogenes* by multiplex PCR. Front. Microbiol. 11:1309. doi: 10.3389/fmicb.2020.01309, PMID: 32676058 PMC7333235

[ref12] Fox E. M. Allnutt T. Bradbury M. I. Fanning S. Chandry P. S. (2016). Comparative genomics of the *Listeria monocytogenes* ST204 subgroup. Front. Microbiol. 7:2057. doi: 10.3389/fmicb.2016.02057, PMID: 28066377 PMC5177744

[ref13] Gupta P. Adhikari A. (2022). Novel approaches to environmental monitoring and control of *Listeria monocytogenes* in food production facilities. Food Secur. 11:1760. doi: 10.3390/foods11121760, PMID: 35741961 PMC9222551

[ref14] He P. Wang H. Yan Y. Zhu G. Chen Z. (2022). Development and application of a multiplex fluorescent PCR for *Shigella* detection and species identification. J. Fluoresc. 32, 707–713. doi: 10.1007/s10895-021-02876-0, PMID: 35044573

[ref15] Hein I. Klinger S. Dooms M. Flekna G. Stessl B. Leclercq A.. (2011). Stress survival islet 1 (SSI-1) survey in *Listeria monocytogenes* reveals an insert common to *listeria innocua* in sequence type 121 *L. monocytogenes* strains. Appl. Environ. Microbiol. 77, 2169–2173. doi: 10.1128/AEM.02159-10, PMID: 21239547 PMC3067325

[ref16] Henri C. Félix B. Guillier L. Leekitcharoenphon P. Michelon D. Mariet J. F.. (2016). Population genetic structure of *Listeria monocytogenes* strains as determined by pulsed-field gel electrophoresis and multilocus sequence typing. Appl. Environ. Microbiol. 82, 5720–5728. doi: 10.1128/AEM.00583-16, PMID: 27235443 PMC5007763

[ref17] Lakicevic B. Jankovic V. Pietzka A. Ruppitsch W. (2023). Wholegenome sequencing as the gold standard approach for control of *Listeria monocytogenes* in the food chain. J. Food Prot. 86:100003. doi: 10.1016/j.jfp.2022.10.002, PMID: 36916580

[ref18] Law J. W. Ab M. N. S. Chan K. G. Lee L. H. (2015). An insight into the isolation, enumeration, and molecular detection of *Listeria monocytogenes* in food. Front. Microbiol. 6:1227. doi: 10.3389/fmicb.2015.01227, PMID: 26579116 PMC4630303

[ref19] Le M. A. Abachin E. Beretti J. L. Berche P. Kayal S. (2011). Diagnosis of *Listeria monocytogenes* meningoencephalitis by real-time PCR for the hly gene. J. Clin. Microbiol. 49, 3917–3923. doi: 10.1128/JCM.01072-11, PMID: 21918022 PMC3209115

[ref20] Lee B. H. Cole S. Badel-Berchoux S. Guillier L. Felix B. Krezdorn N.. (2019). Biofilm formation of *Listeria monocytogenes* strains under food processing environments and Pan-genome-wide association study. Front. Microbiol. 10:2698. doi: 10.3389/fmicb.2019.02698, PMID: 31824466 PMC6882377

[ref21] Li F. Ye Q. Chen M. Zhang J. Xue L. Wang J.. (2021). Multiplex PCR for the identification of pathogenic *Listeria* in Flammulina velutipes plant based on novel specific targets revealed by Pan-genome analysis. Front. Microbiol. 11:634255. doi: 10.3389/fmicb.2020.634255, PMID: 33519795 PMC7843925

[ref22] Liu D. (2013). Molecular approaches to the identification of pathogenic and nonpathogenic listeriae. Microbiol. Insights 6, 59–69. doi: 10.4137/MBI.S10880, PMID: 24826075 PMC3987759

[ref23] Liu S. Liu H. Wang X. Shi L. (2024). The immune system of prokaryotes: potential applications and implications for gene editing. Biotechnol. J. 19:e2300352. doi: 10.1002/biot.202300352, PMID: 38403433

[ref24] Lu Q. Zhu X. Long Q. Yi X. Yang A. Long X.. (2022). Comparative genomics reveal the utilization ability of variable carbohydrates as key genetic features of *Listeria* pathogens in their pathogenic lifestyles. Pathogens 11:1430. doi: 10.3390/pathogens11121430, PMID: 36558765 PMC9784484

[ref25] Mafuna T. Matle I. Magwedere K. Pierneef R. E. Reva O. N. (2021). Whole genome-based characterization of *Listeria monocytogenes* isolates recovered from the food chain in South Africa. Front. Microbiol. 12:669287. doi: 10.3389/fmicb.2021.669287, PMID: 34276601 PMC8283694

[ref26] Mafuna T. Matle I. Magwedere K. Pierneef R. E. Reva O. N. (2022). Comparative genomics of *Listeria* species recovered from meat and food processing facilities. Microbiol. Spectr. 10:e0118922. doi: 10.1128/spectrum.01189-22, PMID: 36066257 PMC9604131

[ref27] Manqele A. Adesiyun A. Mafuna T. Pierneef R. Moerane R. Gcebe N. (2024). Virulence potential and antimicrobial resistance of *Listeria monocytogenes* isolates obtained from beef and beef-based products deciphered using whole-genome sequencing. Microorganisms 12:1166. doi: 10.3390/microorganisms12061166, PMID: 38930548 PMC11205329

[ref28] Matle I. Mbatha K. R. Madoroba E. (2020). A review of *Listeria monocytogenes* from meat and meat products: epidemiology, virulence factors, antimicrobial resistance and diagnosis. Onderstepoort J. Vet. Res. 87, e1–e20. doi: 10.4102/ojvr.v87i1.1869, PMID: 33054262 PMC7565150

[ref29] Montero D. Bodero M. Riveros G. Lapierre L. Gaggero A. Vidal R. M.. (2015). Molecular epidemiology and genetic diversity of *Listeria monocytogenes* isolates from a wide variety of ready-to-eat foods and their relationship to clinical strains from listeriosis outbreaks in Chile. Front. Microbiol. 6:384. doi: 10.3389/fmicb.2015.00384, PMID: 25983727 PMC4415432

[ref30] Nwaiwu O. (2022). Comparative genome analysis of the first *Listeria monocytogenes* core genome multi-locus sequence types CT2050 AND CT2051 strains with their close relatives. AIMS Microbiol. 8, 61–72. doi: 10.3934/microbiol.2022006, PMID: 35496987 PMC8995181

[ref31] Osek J. Wieczorek K. (2022). *Listeria monocytogenes*-how this pathogen uses its virulence mechanisms to infect the hosts. Pathogens 11:1491. doi: 10.3390/pathogens11121491, PMID: 36558825 PMC9783847

[ref32] Page A. J. Cummins C. A. Hunt M. Wong V. K. Reuter S. Holden M. T.. (2015). Roary: rapid large-scale prokaryote pan genome analysis. Bioinformatics 31, 3691–3693. doi: 10.1093/bioinformatics/btv421, PMID: 26198102 PMC4817141

[ref33] Pang R. Xie T. Wu Q. Li Y. Lei T. Zhang J.. (2019). Comparative genomic analysis reveals the potential risk of *Vibrio parahaemolyticus* isolated from ready-to-eat foods in China. Front. Microbiol. 10:186. doi: 10.3389/fmicb.2019.00186, PMID: 30792709 PMC6374323

[ref34] Seemann T. (2014). Prokka: rapid prokaryotic genome annotation. Bioinformatics 30, 2068–2069. doi: 10.1093/bioinformatics/btu153, PMID: 24642063

[ref35] Sloan A. Wang G. Cheng K. (2017). Traditional approaches versus mass spectrometry in bacterial identification and typing. Clin. Chim. Acta 473, 180–185. doi: 10.1016/j.cca.2017.08.035, PMID: 28866114

[ref36] Stessl B. Rückerl I. Wagner M. (2014). Multilocus sequence typing (MLST) of *Listeria monocytogenes*. Methods Mol. Biol. 1157, 73–83. doi: 10.1007/978-1-4939-0703-8_6 24792549

[ref37] Wang Y. Ji Q. Li S. Liu M. (2021). Prevalence and genetic diversity of *Listeria monocytogenes* isolated from retail pork in Wuhan, China. Front. Microbiol. 12:620482. doi: 10.3389/fmicb.2021.620482, PMID: 33767677 PMC7986423

[ref38] Wu S. Wu Q. Zhang J. Chen M. Guo W. (2016). Analysis of multilocus sequence typing and virulence characterization of *Listeria monocytogenes* isolates from Chinese retail ready-to-eat food. Front. Microbiol. 7:168. doi: 10.3389/fmicb.2016.00168, PMID: 26909076 PMC4754575

[ref39] Zhang J. Cao G. Xu X. Allard M. Li P. Brown E. (2016). Evolution and diversity of *Listeria monocytogenes* from clinical and food samples in Shanghai, China. Front. Microbiol. 7:1138. doi: 10.3389/fmicb.2016.01138, PMID: 27499751 PMC4956650

[ref40] Zhang B. Ren H. Wang X. Han C. Jin Y. Hu X.. (2024). Comparative genomics analysis to explore the biodiversity and mining novel target genes of *Listeria monocytogenes* strains from different regions. Front. Microbiol. 15:1424868. doi: 10.3389/fmicb.2024.1424868, PMID: 38962128 PMC11220162

[ref41] Zhang H. Wang J. Chang Z. Liu X. Chen W. Yu Y.. (2021). *Listeria monocytogenes* contamination characteristics in two ready-to-eat meat plants from 2019 to 2020 in Shanghai. Front. Microbiol. 12:729114. doi: 10.3389/fmicb.2021.729114, PMID: 34512606 PMC8427505

[ref42] Zhu L. Ji X. Wu Y. Xu W. Wang F. Huang X. (2023). Molecular characterization of *Listeria monocytogenes* strains isolated from imported food in China from 14 countries/regions, 2003-2018. Front. Cell. Infect. Microbiol. 13:1287564. doi: 10.3389/fcimb.2023.1287564, PMID: 38179422 PMC10765603

