# Supplementary material for: Exploration of the biodiversity and mining novel target genes of Listeria monocytogenes strains isolated from beef through comparative genomics analysis
Source: Front Microbiol. 2025 Apr 28;16:1560974. doi: 10.3389/fmicb.2025.1560974 (PMC12066634; doi:10.3389/fmicb.2025.1560974)
Supplement: Supplementary file 1 [file Data_Sheet_1.pdf]

Supplementary Material

Exploration of the biodiversity and mining novel target genes of *Listeria monocytogenes* strains isolated from beef through comparative genomics analysis

Bo Zhang[1†], Wenjie Sun[1†], Xiaoxu Wang[2†], Honglin Ren[1†], Yang Wang[1], Shaohui Hu[1], Chengwei Li[1], Yuzhu Wang[1], Jiaqi Hou[1], Xueyu Hu[1], Ruoran Shi[1], Yansong Li[1], Shiying Lu[1], Qiang Lu[1], Zengshan Liu[1] and Pan Hu[1*]

[1]State Key Laboratory for Diagnosis and Treatment of Severe Zoonotic Infectious Diseases, Key Laboratory for Zoonosis Research of the Ministry of Education, Institute of Zoonosis, and College of Veterinary Medicine, Jilin University, Changchun 130062, China

[2]Institute of Special Animal and Plant Sciences of Chinese Academy of Agricultural Sciences, Changchun 130112, Jilin, China

***Corresponding author:**

Pan Hu

E-mail: hupan84@163.com

Telephone: +86 15004318401

[†]These authors contributed equally to this work and share first authorship.

Table S1. Detailed information on Genbank accession numbers, strain names, genome size, GC content, number of contigs and N50 of *L. monocytogenes* strains isolated from beef.

Genbank	Strain names	Genome size(pMb)	GC content(%)	No. of contigs	N50(Kbp)	Genbank	Strain names	Genome size(pMb)	GC content(%)	No. of contigs	N50(Kbp)
GCA_003002735.1	CFSAN035200	3	38	19	477.7	GCA_030960545.1	L01500	3.1	38	20	430.3
GCA_004667505.1	CFSAN048830	3.1	38	26	263.4	GCA_030975745.1	L01501	3.5	38	22	483
GCA_023573915.1	JW-2022B	3	38	14	546	GCA_030963945.1	L01583	3.2	38	22	398.4
GCA_023573925.1	JW-2022D	3	38	17	457.8	GCA_030975765.1	L01584	3	38	16	510.2
GCA_023574005.1	JW-2022E	3	38	13	497.4	GCA_030972235.1	L01587	3	38	20	476.5
GCA_023574035.1	JW-2022F	3	38	19	477.9	GCA_030963905.1	L01592	3.2	38	21	462.2
GCA_030989305.1	L00363	3.1	38	23	487	GCA_030969265.1	L01598	3.1	38	17	477.2
GCA_030977345.1	L00369	3	38	19	425.3	GCA_030963865.1	L01599	3.1	38	16	585.2
GCA_030968765.1	L00372	3.1	38	18	397.7	GCA_030960485.1	L01605	3.2	38	15	476.9
GCA_030989225.1	L00389	3.1	37.5	29	245.1	GCA_030969685.1	L01607	3.2	38	22	532.5
GCA_030975385.1	L00407	3.4	38	31	482.6	GCA_030963885.1	L01610	3.1	38	17	476.2
GCA_030968705.1	L00412	3.1	38	20	298.7	GCA_030960465.1	L01613	3.2	38	16	503.9
GCA_030968685.1	L00417	3	38	18	434.7	GCA_030960425.1	L01617	3.1	38	23	476.5
GCA_030978765.1	L00431	3.1	38	17	268.5	GCA_030963845.1	L01619	3.1	38	18	476.5
GCA_030975325.1	L00432	3	38	15	476.6	GCA_030963645.1	L01621	3.2	38	19	476.2
GCA_030978745.1	L00438	3.2	38	19	477.4	GCA_030963625.1	L01646	3.2	38	19	476.2
GCA_030978705.1	L00444	3.1	38	23	564.5	GCA_030963565.1	L01716	3	38	15	434.7
GCA_030978685.1	L00450	3	38	18	526	GCA_030963545.1	L01733	3	38	19	541.7
GCA_030977225.1	L00452	3.1	38	21	566.7	GCA_030975725.1	L01750	3.2	38	18	476.2
GCA_030978645.1	L00457	3.1	38	18	429.4	GCA_030960405.1	L01771	3.1	38	34	529.4
GCA_030977145.1	L00458	3.1	38	20	369.1	GCA_030975665.1	L01795	3	38	15	1500
GCA_030975345.1	L00465	3.1	38	32	475.8	GCA_030963105.1	L01799	3.1	38	23	542.3
GCA_030975305.1	L00471	3	38	15	476.6	GCA_030975645.1	L01800	3	38	15	1500
GCA_030968585.1	L00474	3.1	38	26	308.1	GCA_030963505.1	L01810	3.1	38	20	476.7
GCA_030977125.1	L00478	3	38	16	476.6	GCA_030969665.1	L01835	3	38	15	581.7
GCA_030968465.1	L00482	3.1	38	24	331.3	GCA_030975605.1	L01851	3.1	38	20	475.1
GCA_030982575.1	L00487	3.2	38	28	476.1	GCA_022683845.1	L1106	3	38	46	345.2
GCA_030977065.1	L00489	3.2	37.5	20	476.4	GCA_022683815.1	L1184	3	38	40	369.8
GCA_030978525.1	L00491	3.1	38	26	291.7	GCA_022683735.1	L1330	3	38	43	398
GCA_030978465.1	L00493	3	38	16	514.9	GCA_022683715.1	L1336	3	38	53	295.5
GCA_030975265.1	L00496	3.1	38	25	477.2	GCA_022683675.1	L1381	3	38	49	312
GCA_030977025.1	L00508	3.1	38	22	566.6	GCA_022683695.1	L1393	3.1	38	51	314.7
GCA_030968405.1	L00533	3.1	38	19	567.4	GCA_022684395.1	L1598	3	38	35	542.5
GCA_030968345.1	L00545	3.1	38	22	564	GCA_022684255.1	L1608	3	38	15	478.7
GCA_030975225.1	L00557	3	38	20	425.3	GCA_022684535.1	L1661	3	38	16	568.6
GCA_030978405.1	L00574	3.2	38	18	524.5	GCA_027980745.1	L17	3	38	14	477.7
GCA_030976985.1	L00578	3.5	37.5	65	238	GCA_022684495.1	L1756	3	37.5	18	399.3
GCA_030976965.1	L00596	3.1	38	15	476.3	GCA_022684215.1	L1778	3	38	17	435.7
GCA_030968285.1	L00597	3	38	17	300.1	GCA_027981075.1	L18	3	38	13	477.7
GCA_030978445.1	L00599	3.1	38	30	477.2	GCA_022684225.1	L1861	3	38	28	330.1
GCA_030978425.1	L00613	3.2	38	20	566.6	GCA_022684175.1	L2099	3	37.5	18	399.3
GCA_030975175.1	L00618	3.2	38	20	566.2	GCA_022684155.1	L2119	2.9	38	22	480.6
GCA_030968205.1	L00634	3	38	20	430.2	GCA_022684065.1	L2124	2.9	38	18	480.6
GCA_030975145.1	L00636	3.2	38	25	434.9	GCA_022684115.1	L2128	2.9	38	27	480.6
GCA_030968325.1	L00638	3.1	38	18	438.5	GCA_022684135.1	L2131	2.9	38	15	480.6
GCA_030978325.1	L00641	3.3	38	21	420.3	GCA_022684315.1	L2132	2.9	38	28	480.6
GCA_030978305.1	L00642	3.1	38	22	477.2	GCA_022684295.1	L2146	3	38	45	319.2
GCA_030976885.1	L00646	2.9	38	16	566.7	GCA_022684445.1	L2185	3	38	20	308.1
GCA_030978265.1	L00649	3.1	38	21	566.7	GCA_027982115.1	L22	3	38	16	480
GCA_030968185.1	L00654	3	38	14	433.8	GCA_027982085.1	L40	2.9	38	11	527.8
GCA_030967565.1	L00668	3.1	38	26	477.2	GCA_027982205.1	L41	2.9	38	14	468.5
GCA_030967515.1	L00679	3.1	38	21	477.2	GCA_022683605.1	L431	3	38	60	319.4
GCA_030978065.1	L00700	3.1	38	20	476.8	GCA_027982145.1	L44	3.1	37.5	22	395
GCA_030976765.1	L00702	3.6	38	24	448.8	GCA_027982235.1	L53	3	38	14	477.7
GCA_030967345.1	L00715	3.2	38	25	434.9	GCA_022683035.1	L867	3	38	28	528.7
GCA_030989705.1	L00723	3.2	38	20	430.2	GCA_003191365.1	LM-F-24	2.9	38	14	735.3
GCA_030967265.1	L00728	3	38	18	438.5	GCA_003191285.1	LM-F-25	3.1	38	42	193.9
GCA_030967165.1	L00741	3.1	38	51	389.8	GCA_003190965.1	LM-F-31	3	38	6	588.6
GCA_030977925.1	L00745	3.2	38	24	434.9	GCA_022682455.1	LYJ24890	3.1	38	64	317.2
GCA_030967065.1	L00752	3.2	38	19	430.2	GCA_015525835.1	OSF107796	3	38	46	130.4
GCA_030977885.1	L00762	3	38	17	617.3	GCA_015527105.1	OSF107798	3	38	63	87.4
GCA_030974965.1	L00781	3	38	19	617.3	GCA_015527285.1	OSF107815	3.1	38	64	86.1
GCA_030966985.1	L00801	3.2	38	16	509.6	GCA_015961325.1	OSF107894	3	38	108	56.3
GCA_030976425.1	L00802	3.2	38	24	477.2	GCA_015961605.1	OSF107898	2.9	38	128	41.5
GCA_030977745.1	L00812	3	38	20	337.8	GCA_015961305.1	OSF107908	2.9	38	113	70.8
GCA_030966885.1	L00814	3.2	38	25	448.3	GCA_015961965.1	OSF107910	2.9	38	104	66.1
GCA_030974905.1	L00819	3.1	38	25	477.2	GCA_015962185.1	OSF107914	2.9	38	81	105.1
GCA_030974925.1	L00823	3	38	18	450	GCA_015961165.1	OSF107921	3	38	124	41.5
GCA_030977665.1	L00825	3.2	38	18	507.9	GCA_015962575.1	OSF107924	2.9	38	91	59.2
GCA_030977525.1	L00872	3.2	38	31	462.1	GCA_015963205.1	OSF107925	3	38	87	67.4
GCA_030976285.1	L00881	3.2	38	22	434.9	GCA_015963165.1	OSF107930	2.9	38	83	78.5
GCA_030962805.1	L00887	3.1	38	25	354.7	GCA_015963025.1	OSF107934	2.9	38	113	64
GCA_030962745.1	L00894	3	38	18	437.1	GCA_015842075.1	OSF107943	2.9	38	122	53.1
GCA_030963365.1	L00919	3	38	15	548.6	GCA_015882185.1	OSF107953	2.9	38	101	358.1
GCA_030974625.1	L00920	3.2	38	24	448.7	GCA_015526265.1	OSF107984	3	38	106	63.2
GCA_030976155.1	L00929	3.2	38	17	507.7	GCA_015526905.1	OSF107989	3	38	151	39.8
GCA_030974585.1	L00930	3.1	38	22	565.4	GCA_015527225.1	OSF107990	2.9	38	107	63.3
GCA_030976085.1	L00936	3.1	38	23	238.1	GCA_015526285.1	OSF107995	3	38	135	45.2
GCA_030974545.1	L00941	3.1	38	17	579.5	GCA_015526605.1	OSF107996	3	38	178	31.3
GCA_030969505.1	L00943	3	38	16	505.5	GCA_015526535.1	OSF107998	2.9	38	176	32.7
GCA_030962605.1	L00946	3.1	38	24	509.4	GCA_015526205.1	OSF107999	2.9	38	151	37.6
GCA_030962525.1	L00947	3.1	38	20	524.5	GCA_015526005.1	OSF108000	2.9	38	88	73.1
GCA_030971885.1	L00958	3.2	38	15	507.9	GCA_015533895.1	OSF108007	2.9	38	82	62.8
GCA_030962505.1	L00959	3	38	20	356.6	GCA_015525445.1	OSF108010	3.1	38	121	63.3
GCA_030966305.1	L00960	3.1	38	21	508.9	GCA_015535295.1	OSF108026	3	38	79	89.6
GCA_030976045.1	L00973	3	38	17	476.2	GCA_015564535.1	OSF108046	3	38	125	54.3
GCA_030971865.1	L00981	3.1	38	21	527.6	GCA_015525455.1	OSF108055	3	38	93	69.5

GCA_030974565.1	L00982	3.2	38	21	527.6	GCA_015534915.1	OSF108088	2.9	38	50	117
GCA_030971745.1	L00988	3.1	37.5	21	527.7	GCA_015535115.1	OSF108091	2.9	38	74	66.5
GCA_030965605.1	L01004	3.1	38	23	477.2	GCA_015534075.1	OSF108092	2.9	38	50	154.7
GCA_030961485.1	L01009	3	38	20	434.7	GCA_015533815.1	OSF108096	2.9	38	30	215.1
GCA_030971695.1	L01012	3.1	37.5	22	477.7	GCA_016448785.1	OSF108108	2.9	38	66	91.6
GCA_030965545.1	L01019	3.4	38	29	357.6	GCA_015539425.1	OSF108110	2.9	38	47	115.7
GCA_030974505.1	L01023	3.1	38	18	567.5	GCA_015561785.1	OSF108142	3	38	95	74.6
GCA_030962465.1	L01025	3.1	38	17	567.5	GCA_015536245.1	OSF108148	3	38	155	37
GCA_030962445.1	L01026	3	38	18	552.2	GCA_015535895.1	OSF108152	3	38	112	64.5
GCA_030965515.1	L01030	3.2	38	34	435.4	GCA_015534475.1	OSF108172	2.9	38	75	79.9
GCA_030963405.1	L01038	3.2	38	18	507.9	GCA_015533545.1	OSF108183	2.9	38	78	82.5
GCA_030974435.1	L01041	3.1	38	22	564.6	GCA_015564835.1	OSF108193	3	38	312	33.4
GCA_030962405.1	L01042	3.1	38	19	477.2	GCA_015563085.1	OSF108201	2.9	38	84	72.8
GCA_030974425.1	L01045	3.1	38	23	450	GCA_015542345.1	OSF108217	2.9	38	53	152.9
GCA_030974405.1	L01049	3.3	38	33	267.5	GCA_015563705.1	OSF108226	2.9	38	37	182.5
GCA_030974385.1	L01050	3.2	38	34	308.1	GCA_015535935.1	OSF108260	3	38	93	65.6
GCA_030963415.1	L01053	3	38	16	565.8	GCA_015534405.1	OSF108274	3	38	103	64
GCA_030971115.1	L01054	3	38	27	502.2	GCA_015531045.1	OSF108278	2.9	38	88	68.7
GCA_030971055.1	L01065	3.2	38	18	564.7	GCA_015533505.1	OSF108281	2.9	38	96	70.6
GCA_030962365.1	L01071	3.3	38	23	473.3	GCA_015564515.1	OSF108298	3	38	213	33.3
GCA_030962325.1	L01074	3.4	38	54	160.3	GCA_015539625.1	OSF108309	3	38	106	63.9
GCA_030974325.1	L01075	3.1	38	25	494.7	GCA_015565595.1	OSF108310	2.9	38	151	41.7
GCA_030965465.1	L01076	3.2	38	31	435.4	GCA_015542305.1	OSF108315	2.9	38	18	504
GCA_030974305.1	L01078	3.2	38	15	508.3	GCA_015561645.1	OSF108316	2.9	38	114	55.4
GCA_030970905.1	L01090	3	38	13	524.7	GCA_015564675.1	OSF108323	2.9	38	167	33.4
GCA_030970825.1	L01091	3	38	16	450	GCA_015563165.1	OSF108351	3	38	96	68.4
GCA_030970865.1	L01093	3.2	38	17	507.9	GCA_015562295.1	OSF108357	2.9	38	244	27.2
GCA_030962305.1	L01096	3.4	38	50	192.9	GCA_015565415.1	OSF108376	2.9	38	199	29.3
GCA_030970725.1	L01100	3.1	38	13	524.8	GCA_015564115.1	OSF108389	3	38	221	26.7
GCA_030961345.1	L01108	2.9	38	18	434.7	GCA_015565775.1	OSF108433	3	38	246	24.4
GCA_030962245.1	L01112	3.1	38	24	477.2	GCA_015963225.1	OSF108443	2.9	38	195	28.8
GCA_030974205.1	L01115	3.2	38	17	507.9	GCA_015584475.1	OSF108457	3	38	82	82.3
GCA_030970685.1	L01116	3	38	14	524.7	GCA_015583335.1	OSF108461	2.9	38	136	45
GCA_030965425.1	L01120	3.4	38	32	357.7	GCA_015584735.1	OSF108466	2.9	38	115	47.5
GCA_030974245.1	L01122	3.1	38	13	524.8	GCA_015962665.1	OSF108467	2.9	38	134	41.5
GCA_030962225.1	L01123	3	38	18	331.2	GCA_015599325.1	OSF108470	3	38	139	43.7
GCA_030962205.1	L01128	3.1	38	19	477.2	GCA_015595725.1	OSF108498	3	38	98	56
GCA_030964725.1	L01132	3	38	21	543.1	GCA_015961665.1	OSF108569	3	38	111	49.8
GCA_030964665.1	L01135	3.1	38	20	382.9	GCA_015961585.1	OSF108570	2.9	38	116	54
GCA_030973805.1	L01137	3.3	38	34	530.3	GCA_015961195.1	OSF108572	3	38	149	38.3
GCA_030964625.1	L01138	2.9	38	16	1500	GCA_015962765.1	OSF108581	2.9	38	115	56
GCA_030970085.1	L01140	3	38	14	476.3	GCA_015583085.1	OSF108595	3	38	100	60.7
GCA_030969385.1	L01142	3.1	38	15	476.5	GCA_015583035.1	OSF108608	2.9	38	149	41.5
GCA_030969365.1	L01144	3.1	38	13	542.2	GCA_015842295.1	OSF108930	2.9	38	108	64.2
GCA_030969345.1	L01152	3.1	38	22	543.1	GCA_015841145.1	OSF108960	2.9	38	60	99.7
GCA_030973965.1	L01153	3	38	11	542.2	GCA_015842055.1	OSF108966	2.9	38	47	126.6
GCA_030964605.1	L01154	3.1	38	41	477.2	GCA_015842415.1	OSF108976	3	38	36	212.2
GCA_030964485.1	L01164	3	38	18	584.4	GCA_018104005.1	OSF111252	3.1	38	42	182.4
GCA_030960985.1	L01166	3.1	38	21	477.2	GCA_018103075.1	OSF111260	3	38	169	30.4
GCA_030973745.1	L01168	3.1	38	28	511	GCA_018103095.1	OSF111269	3	38	198	26.6
GCA_030961165.1	L01180	3.1	38	24	477.2	GCA_018103835.1	OSF111275	3	38	183	30.7
GCA_030970045.1	L01184	3.2	38	75	477.2	GCA_018103675.1	OSF111283	2.9	38	34	201.9
GCA_030961145.1	L01185	3.1	38	19	550.6	GCA_018104385.1	OSF111286	3	38	85	80.1
GCA_030964525.1	L01187	3.1	38	21	582.7	GCA_018103995.1	OSF111300	3	38	218	25.8
GCA_030964445.1	L01191	3.1	38	20	477.2	GCA_018103795.1	OSF111301	3	38	214	28.2
GCA_030961105.1	L01193	3	38	20	259.2	GCA_018103595.1	OSF111302	2.9	38	159	35.9
GCA_030970025.1	L01194	3.4	38	54	160.3	GCA_018104545.1	OSF111307	2.9	38	104	58
GCA_030961085.1	L01199	3.1	38	20	477.2	GCA_018104155.1	OSF111308	3	38	35	277.7
GCA_030964385.1	L01219	3	38	19	550	GCA_020803475.1	PNUSAL012100	3	38	20	580.8
GCA_030969945.1	L01223	3.1	38	18	450.3	GCA_020803995.1	PNUSAL012101	3	38	14	580.9
GCA_030969925.1	L01227	3.1	38	29	554.8	GCA_020804135.1	PNUSAL012102	3	38	15	563
GCA_030975965.1	L01233	3.1	38	28	511.6	GCA_020803755.1	PNUSAL012103	3.2	38	22	380.6
GCA_030975945.1	L01240	3.1	38	26	339.1	GCA_020804155.1	PNUSAL012104	3.1	38	24	475.2
GCA_030973665.1	L01241	3	38	21	450	GCA_021528855.1	PNUSAL012900	3.2	38	22	476.2
GCA_030972775.1	L01251	3.1	38	17	584.8	GCA_021546375.1	PNUSAL012909	3	38	19	477.2
GCA_030969885.1	L01264	3	38	39	477.2	GCA_021546295.1	PNUSAL012910	3.1	38	22	299.4
GCA_030969845.1	L01271	3.1	38	29	477.2	GCA_022628835.1	PNUSAL013433	3	38	15	1500
GCA_030972795.1	L01288	3.1	38	24	434.7	GCA_023270935.1	PNUSAL013434	3.2	38	21	476.2
GCA_030960925.1	L01299	3.6	37.5	38	367.4	GCA_022657505.1	PNUSAL013435	3	38	21	544.6
GCA_030972645.1	L01304	3.2	38	29	476.9	GCA_022657475.1	PNUSAL013436	3.1	38	20	476.3
GCA_030972605.1	L01305	3.1	38	30	434.9	GCA_022629855.1	PNUSAL013437	3.1	38	23	508
GCA_030964125.1	L01373	3	38	25	497.3	GCA_022657495.1	PNUSAL013438	3.2	38	24	503.9
GCA_030964085.1	L01393	3.1	38	28	511	GCA_022629015.1	PNUSAL013439	3.2	38	22	476.2
GCA_030960785.1	L01394	3.1	38	18	584	GCA_024849965.1	PNUSAL014862	3.2	38	24	477.2
GCA_030972445.1	L01419	2.9	38	23	449.9	GCA_024849945.1	PNUSAL014863	3.2	38	23	479.6
GCA_030960705.1	L01434	3	38	32	477.2	GCA_024850005.1	PNUSAL014864	3.1	38	23	503.6
GCA_030969765.1	L01435	3.1	38	26	476.8	GCA_025310455.1	PNUSAL015301	3.1	38	22	503.5
GCA_030975825.1	L01461	3	38	18	450	GCA_025264765.1	PNUSAL015302	3.1	38	21	503.6
GCA_030960645.1	L01469	3.1	38	23	432.2	GCA_025311335.1	PNUSAL015303	3.1	38	20	503.5
GCA_030960585.1	L01470	3	38	13	495.8	GCA_025413215.1	PNUSAL015360	3.2	38	18	503.6
GCA_030972365.1	L01486	3	38	23	345.7	GCA_025415875.1	PNUSAL015361	3.2	38	16	508.5
GCA_030975805.1	L01488	3.2	38	24	398.4	GCA_025414895.1	PNUSAL015362	3.3	38	21	349.4
GCA_030963965.1	L01490	3.1	38	23	336.9	GCA_025412545.1	PNUSAL015363	3.2	38	20	503.9
GCA_030960605.1	L01495	3	38	15	476.3	GCA_040551615.1	PNUSAL022259	3.3	38	27	476.3
GCA_030963145.1	L01499	3.1	38	22	339.1	GCA_013415135.1	SCPM-O-B-8838	2.9	38	43	168.1

Table S2. Detailed information of *L. monocytogenes* strains from beef, other *Listeria* strains, and *non-Listeria* strains.

Bacterial species	Genbank				Genome size (Mbp)	GC content(%)	No. of contigs	N50(Kbp)	Isolation Source
	GCA_003002735.1	GCA_030971865.1	GCA_030960545.1	GCA_015525455.1					
	GCA_004667505.1	GCA_030974565.1	GCA_030975745.1	GCA_015534915.1					
	GCA_023573915.1	GCA_030971745.1	GCA_030963945.1	GCA_015535115.1					
	GCA_023573925.1	GCA_030965605.1	GCA_030975765.1	GCA_015534075.1					
	GCA_023574005.1	GCA_030961485.1	GCA_030972235.1	GCA_015533815.1					
	GCA_023574035.1	GCA_030971695.1	GCA_030963905.1	GCA_016448785.1					
	GCA_030989305.1	GCA_030965545.1	GCA_030969265.1	GCA_015539425.1					
	GCA_030977345.1	GCA_030974505.1	GCA_030963865.1	GCA_015561785.1					
	GCA_030968765.1	GCA_030962465.1	GCA_030960485.1	GCA_015536245.1					
	GCA_030989225.1	GCA_030962445.1	GCA_030969685.1	GCA_015535895.1					
	GCA_030975385.1	GCA_030965515.1	GCA_030963885.1	GCA_015534475.1					
	GCA_030968705.1	GCA_030963405.1	GCA_030960465.1	GCA_015533545.1					
	GCA_030968685.1	GCA_030974435.1	GCA_030960425.1	GCA_015564835.1					
	GCA_030978765.1	GCA_030962405.1	GCA_030963845.1	GCA_015563085.1					
	GCA_030975325.1	GCA_030974425.1	GCA_030963645.1	GCA_015542345.1					
	GCA_030978745.1	GCA_030974405.1	GCA_030963625.1	GCA_015563705.1					
	GCA_030978705.1	GCA_030974385.1	GCA_030963565.1	GCA_015535935.1					
	GCA_030978685.1	GCA_030963415.1	GCA_030963545.1	GCA_015534405.1					
	GCA_030977225.1	GCA_030971115.1	GCA_030975725.1	GCA_015531045.1					
	GCA_030978645.1	GCA_030971055.1	GCA_030960405.1	GCA_015533505.1					
	GCA_030977145.1	GCA_030962365.1	GCA_030975665.1	GCA_015564515.1					
	GCA_030975345.1	GCA_030962325.1	GCA_030963105.1	GCA_015539625.1					
	GCA_030975305.1	GCA_030974325.1	GCA_030975645.1	GCA_015565595.1					
	GCA_030968585.1	GCA_030965465.1	GCA_030963505.1	GCA_015542305.1					
	GCA_030977125.1	GCA_030974305.1	GCA_030969665.1	GCA_015561645.1					
	GCA_030968465.1	GCA_030970905.1	GCA_030975605.1	GCA_015564675.1					
	GCA_030982575.1	GCA_030970825.1	GCA_022683845.1	GCA_015563165.1					
	GCA_030977065.1	GCA_030970865.1	GCA_022683815.1	GCA_015562295.1					
	GCA_030978525.1	GCA_030962305.1	GCA_022683735.1	GCA_015565415.1					
Listeria monocytogenes	GCA_030978465.1	GCA_030970725.1	GCA_022683715.1	GCA_015564115.1	2.9-3.6	37.5-38	≤312	24.4-1500	beef
	GCA_030975265.1	GCA_030961345.1	GCA_022683675.1	GCA_015565775.1					
	GCA_030977025.1	GCA_030962245.1	GCA_022683695.1	GCA_015963225.1					
	GCA_030968405.1	GCA_030974205.1	GCA_022684395.1	GCA_015584475.1					
	GCA_030968345.1	GCA_030970685.1	GCA_022684255.1	GCA_015583335.1					
	GCA_030975225.1	GCA_030965425.1	GCA_022684535.1	GCA_015584735.1					
	GCA_030978405.1	GCA_030974245.1	GCA_027980745.1	GCA_015962665.1					
	GCA_030976985.1	GCA_030962225.1	GCA_022684495.1	GCA_015599325.1					
	GCA_030976965.1	GCA_030962205.1	GCA_022684215.1	GCA_015595725.1					
	GCA_030968285.1	GCA_030964725.1	GCA_027981075.1	GCA_015961665.1					
	GCA_030978445.1	GCA_030964665.1	GCA_022684225.1	GCA_015961585.1					
	GCA_030978425.1	GCA_030973805.1	GCA_022684175.1	GCA_015961195.1					
	GCA_030975175.1	GCA_030964625.1	GCA_022684155.1	GCA_015962765.1					
	GCA_030968205.1	GCA_030970085.1	GCA_022684065.1	GCA_015583085.1					
	GCA_030975145.1	GCA_030969385.1	GCA_022684115.1	GCA_015583035.1					
	GCA_030968325.1	GCA_030969365.1	GCA_022684135.1	GCA_015842295.1					
	GCA_030978325.1	GCA_030969345.1	GCA_022684315.1	GCA_015841145.1					
	GCA_030978305.1	GCA_030973965.1	GCA_022684295.1	GCA_015842055.1					
	GCA_030976885.1	GCA_030964605.1	GCA_022684445.1	GCA_015842415.1					
	GCA_030978265.1	GCA_030964485.1	GCA_027982115.1	GCA_018104005.1					
	GCA_030968185.1	GCA_030960985.1	GCA_027982085.1	GCA_018103075.1					
	GCA_030967565.1	GCA_030973745.1	GCA_027982205.1	GCA_018103095.1					
	GCA_030967515.1	GCA_030961165.1	GCA_022683605.1	GCA_018103835.1					
	GCA_030978065.1	GCA_030970045.1	GCA_027982145.1	GCA_018103675.1					
	GCA_030976765.1	GCA_030961145.1	GCA_027982235.1	GCA_018104385.1					
	GCA_030967345.1	GCA_030964525.1	GCA_022683035.1	GCA_018103995.1					
	GCA_030989705.1	GCA_030964445.1	GCA_003191365.1	GCA_018103795.1					
	GCA_030967265.1	GCA_030961105.1	GCA_003191285.1	GCA_018103595.1					
	GCA_030967165.1	GCA_030970025.1	GCA_003190965.1	GCA_018104545.1					
	GCA_030977925.1	GCA_030961085.1	GCA_022682455.1	GCA_018104155.1					

	GCA_030967065.1	GCA_030964385.1	GCA_015525835.1	GCA_020803475.1	
	GCA_030977885.1	GCA_030969945.1	GCA_015527105.1	GCA_020803995.1	
	GCA_030974965.1	GCA_030969925.1	GCA_015527285.1	GCA_020804135.1	
	GCA_030966985.1	GCA_030975965.1	GCA_015961325.1	GCA_020803755.1	
	GCA_030976425.1	GCA_030975945.1	GCA_015961605.1	GCA_020804155.1	
	GCA_030977745.1	GCA_030973665.1	GCA_015961305.1	GCA_021528855.1	
	GCA_030966885.1	GCA_030972775.1	GCA_015961965.1	GCA_021546375.1	
	GCA_030974905.1	GCA_030969885.1	GCA_015962185.1	GCA_021546295.1	
	GCA_030974925.1	GCA_030969845.1	GCA_015961165.1	GCA_022628835.1	
	GCA_030977665.1	GCA_030972795.1	GCA_015962575.1	GCA_023270935.1	
	GCA_030977525.1	GCA_030960925.1	GCA_015963205.1	GCA_022657505.1	
	GCA_030976285.1	GCA_030972645.1	GCA_015963165.1	GCA_022657475.1	
	GCA_030962805.1	GCA_030972605.1	GCA_015963025.1	GCA_022629855.1	
	GCA_030962745.1	GCA_030964125.1	GCA_015842075.1	GCA_022657495.1	
	GCA_030963365.1	GCA_030964085.1	GCA_015882185.1	GCA_022629015.1	
	GCA_030974625.1	GCA_030960785.1	GCA_015526265.1	GCA_024849965.1	
	GCA_030976155.1	GCA_030972445.1	GCA_015526905.1	GCA_024849945.1	
	GCA_030974585.1	GCA_030960705.1	GCA_015527225.1	GCA_024850005.1	
	GCA_030976085.1	GCA_030969765.1	GCA_015526285.1	GCA_025310455.1	
	GCA_030974545.1	GCA_030975825.1	GCA_015526605.1	GCA_025264765.1	
	GCA_030969505.1	GCA_030960645.1	GCA_015526535.1	GCA_025311335.1	
	GCA_030962605.1	GCA_030960585.1	GCA_015526205.1	GCA_025413215.1	
	GCA_030962525.1	GCA_030972365.1	GCA_015526005.1	GCA_025415875.1	
	GCA_030971885.1	GCA_030975805.1	GCA_015533895.1	GCA_025414895.1	
	GCA_030962505.1	GCA_030963965.1	GCA_015525445.1	GCA_025412545.1	
	GCA_030966305.1	GCA_030960605.1	GCA_015535295.1	GCA_040551615.1	
	GCA_030976045.1	GCA_030963145.1	GCA_015564535.1	GCA_013415135.1	
Listeria innocua	GCA_009648575.1				Reference genome
Listeria ivanovii	GCA_000763515.1				Reference genome
Listeria welshimeri	GCA_900187315.1				Reference genome
Listeria grayi	GCA_900638015.1				Reference genome
Listeria seeligeri	GCA_000027145.1				Reference genome
Escherichia coli	GCA_000005845.2				Reference genome
Salmonella enterica	GCA_000006945.2				Reference genome
Clostridium tetani E88	GCA_000007625.1				Reference genome
Bacillus anthracis	GCA_000008445.1				Reference genome
Salmonella bongori	GCA_000439255.1				Reference genome
Bacillus cereus	GCA_002220285.1				Reference genome
Corynebacterium diphtheriae	GCA_002843135.1				Reference genome
Total	356				

Table S3. Detailed information on potential target genes of *L. monocytogenes* strains isolated from beef.

Potential target genes	Name of target genes	Potential target genes	Name of target genes	Potential target genes	Name of target genes	Potential target genes	Name of target genes
ylmA	lmo1778	xpt	lmo1885	oleD	lmo1477	group_44787	lmo0926
inlC	inlC	yppE	lmo1890	nadD	lmo1488	group_6348	lmo0945
mcsA	lmo0230	ponA	pbpA	aroE_3	lmo1490	group_3430	lmo0946
ispD	lmo0235	group_10748	lmo1893	trmR	lmo1498	yycB	lmo0947
ispF	lmo0236	dnaD	dnaD	group_6112	lmo1506	pdhR	lmo0948
yacP	lmo0242	ypmB	lmo1898	rcsC_1	lmo1508	group_3431	lmo0950
group_3895	lmo0273	panB	panB	iscS_2	lmo1513	group_11188	lmo0951
group_10646	lmo0274	ypjD	lmo1908	group_6118	lmo1518	group_6351	lmo0952
comEC_2	lmo0275	dgcT	lmo1911	hisS	hisS	group_4591	lmo0954
yitU_1	lmo0276	group_10753	lmo1912	dtd	lmo1522	rlhA	lmo0960
group_623	lmo0281	pdeG	lmo1914	group_3265	lmo1525	group_4595	lmo0969
bacF	lmo0286	mleS	lmo1915	group_6142	lmo1526	dltD	dltD
yycH	lmo0289	group_5732	lmo1920	group_6148	lmo1528	group_4598	lmo0976
yycI	lmo0290	aroB	aroB	glpF_1	lmo1539	group_6356	lmo0977
thiE	lmo0318	ndk	ndk	minD	minD	drrA	lmo0979
group_1301	lmo0326	group_2910	lmo1935	hemC	hemC	group_6358	lmo0985
group_808	lmo0334	cmk	cmk	hemA	hemA	group_2462	lmo0986
idi	lmo0368	betI_1	lmo1962	mutM	mutM	group_4599	lmo0987
ung_1	lmo0390	group_10757	lmo1963	argF	argF	crr	lmo1017
group_10661	lmo0393	group_1401	lmo1982	group_2321	lmo1608	cutC_1	lmo1018
group_3912	lmo0394	deoR	lmo1996	group_2333	lmo1613	aptB	lmo1033
group_3913	lmo0395	glmS_2	lmo1999	murJ_2	lmo1624	glpK_2	lmo1034
group_5633	lmo0403	group_5753	lmo2002	group_11046	lmo1626	mobA	lmo1038
group_10668	lmo0413	yurK	lmo2004	trpA	trpA	modA	lmo1041
pgdA	lmo0415	yajO_2	lmo2005	trpF	trpF	moaE	lmo1044
group_10669	lmo0416	mngB_3	lmo2014	trpC	trpC	moaB	lmo1048
group_2803	lmo0417	group_5758	lmo2031	bcrA_2	lmo1636	ldh_1	lmo1057
group_5637	lmo0419	murG	murG	group_11065	lmo1636	group_6370	lmo1059
qorB	lmo0437	murE	murE	sbcD	lmo1646	group_6372	lmo1065
group_10672	lmo0442	pbpB	pbpB	znuA_1	lmo1671	btuF	lmo1073
group_10677	lmo0451	mdrP_1	lmo2043	ycaD	lmo1682	group_11218	lmo1157
group_2001	lmo0511	tmcAL	lmo2049	group_6250	lmo2591	group_2483	lmo1158
group_3933	lmo0516	coaD	lmo2052	truA	truA	group_3466	lmo1178
group_636	lmo0528	group_4052	lmo2059	sapB	lmo2602	group_6401	lmo1183
MENG	lmo0532	yiiM	lmo2060	apbE	lmo2636	group_6405	lmo1189
group_5657	lmo0542	ycnJ	lmo2062	group_11105	lmo2671	cbiA	cbiA
group_860	lmo0548	cbh	lmo2067	group_4476	lmo2672	cobD_1	lmo1192
group_3941	lmo0550	MroQ	lmo2070	tmk	lmo2693	cysG	lmo1201
hisI	lmo0562	group_5767	lmo2071	hexR	lmo2698	cbiL	cbiL
hisA	hisA	group_6633	lmo2074	xylB	lmo2712	cobQ	cbiP
group_10696	lmo0580	tsaE	lmo2078	group_11123	lmo2713	group_11231	lmo1213
group_3948	lmo0585	group_4058	lmo2080	group_4489	lmo2714	lytG_1	lmo1216
group_2008	lmo0590	crcB_1	lmo2081	lrp	lmo2727	group_6419	lmo1225
group_3951	lmo0591	crcB_2	lmo2082	group_6279	lmo2731	ung_2	lmo1227
group_3953	lmo0600	group_5772	lmo2083	group_3351	lmo2735	yslB	lmo1236
group_10697	lmo0603	group_2943	lmo2098	cobB	lmo2739	group_4689	lmo2368
group_5664	lmo0604	norG	lmo2100	group_505	lmo2740	group_4699	lmo2416
slyA_1	lmo0612	nfrA2	lmo2111	group_4498	lmo2742	fpuC	lmo2429
group_5668	lmo0617	group_10777	lmo2112	cocE	lmo2755	group_4708	lmo2441
group_2848	lmo0623	cdaR	lmo2119	group_11125	lmo2767	group_6457	lmo2462
yycN	lmo0624	malP	lmo2121	bglF_2	lmo2772	group_3514	lmo2465
group_5670	lmo0625	group_4068	lmo2129	group_510	lmo2781	pgcA_2	lmo2475
group_5671	lmo0626	gatY_2	lmo2133	bglK_3	lmo2796	mro	lmo2476
COQ5_1	lmo0637	gatY_3	lmo2134	ulaC_3	lmo2797	galE	galE
zosA	lmo0641	frwD	lmo2136	cbbY	lmo2798	group_6461	lmo2479
fsaA	lmo0643	mngA_3	lmo2137	inlJ	lmo2821	group_6464	lmo2485
ltaS2	lmo0644	group_10780	lmo2139	garK_2	lmo2832	group_11264	lmo2487
catE	lmo0646	yhaP	lmo2140	group_6628	lmo2844	group_4715	lmo2490
group_10707	lmo0654	mshD_2	lmo2141	capA	lmo0017	phoR	phoR

pphA	lmo0655	group_5789	lmo2142	cutC_2	lmo0026	group_6469	lmo2504
group_2856	lmo0657	yjcS_2	sepA	ccpA_2	lmo0031	group_6470	spl
group_1358	lmo0671	iolE	lmo2162	nanK	lmo0032	degV	lmo2514
flgG	lmo0682	group_5795	lmo2164	arcC1	lmo0039	yigZ	lmo2516
group_5691	lmo0704	group_10783	lmo2169	group_6310	lmo0057	group_6472	lmo2517
flgB	lmo0710	ohrR	lmo2200	group_240	lmo0075	tagT	lmo2518
group_10720	lmo0718	group_5800	lmo2203	ada	lmo0076	menC	lmo2520
group_3980	lmo0720	hemH	hemH	group_2431	lmo0078	tagA	lmo2521
ribF_1	lmo0728	group_5806	lmo2214	adhR_3	lmo0083	yocH	lmo2522
group_5697	lmo0731	prsA1_2	lmo2219	iolS	lmo0084	ywle	lmo2540
group_5699	lmo0733	fumC	citG	group_6315	lmo0087	ywlC	lmo2541
group_10727	lmo0752	pbpF	lmo2229	atpD_1	lmo0089	prmC	lmo2542
group_3986	lmo0753	iolI	lmo2234	atpG_1	lmo0091	cpoA	lmo2554
group_1375	lmo0754	aroE_2	lmo2236	atpC_1	lmo0093	group_11281	lmo2555
group_3987	lmo0755	ytrB_2	lmo2240	group_3384	lmo0095	dagK_2	lmo2557
cpdA_1	lmo0763	adaA	lmo2243	group_11164	lmo0100	group_6488	lmo2565
lplJ_2	lmo0764	group_5817	lmo2258	ycnE_1	lmo0102	lipL	lmo2566
group_10728	lmo0775	celD	lmo2259	aes_2	lmo0110	group_6492	lmo2578
bglK_1	lmo0776	group_5819	lmo2262	group_3385	lmo0112	ycnE_2	lmo2579
ybaK	lmo0790	group_2105	lmo1266	group_11166	lmo0113	hssS	lmo2582
group_2888	lmo0819	sipV	lmo1270	group_6325	lmaD	group_44656	lmo1687
mvaA	lmo0825	glpQ	lmo1292	group_3386	lmaC	mutY	lmo1689
group_4000	lmo1800	hflX_2	lmo1296	cph2	lmo0131	yfhP	lmo1690
rsgA_1	lmo1819	group_10906	lmo1334	group_1087	lmo0133	recX_1	lmo1693
prkC	lmo1820	gluP	lmo1337	znuA_2	lmo0153	yokD_1	lmo1708
fmt	fmt	group_4206	lmo1344	group_3391	lmo0156	hcxA	lmo1737
coaBC	lmo1825	comGC	lmo1345	dinG_2	lmo0157	lacC_2	fruB
pyrE	pyrE	gcvT	lmo1348	yidA_4	lmo0158	rsuA	lmo2342
pyrB	pyrB	group_2172	lmo1352	rsbRD	lmo0161	group_3565	lmo2344
rsbRD_1	lmo1842	xseA	lmo1361	group_6335	lmo0166	ssuE	lmo2351
mntA	lmo1847	pta_2	lmo1369	group_4568	lmo0170	group_10074	lmo0186
copZ	lmo1852	fni	lmo1383	fbp	fbp	rnmV	lmo0187
yodJ	lmo1855	group_5989	lmo1392	group_1692	lmo0834	group_11320	lmo0191
yceM	lmo1858	group_10942	lmo1395	uvrC_1	lmo0846	prfA_2	prfA
msrB	lmo1859	group_6001	lmo1400	group_4575	lmo0852	plcA	plcA
group_4017	lmo1862	mvaS	lmo1415	pgcA_1	lmo0865	group_3570	lmo0209
ccpN	lmo1865	group_6027	lmo1416	group_4581	lmo0873	group_3571	lmo0212
group_4020	lmo1868	group_6033	lmo1418	lacF_2	lmo0874	murJ_3	lmo0215
group_5725	lmo1870	yjbR	lmo1430	group_4583	lmo0879	divIC	lmo0217
asd	lmo1437	dapA	lmo1435	group_6341	lmo0882	tilS	lmo0219
ispG	lmo1441	lysC_1	lmo1436	group_11184	lmo0899	folP	sul
ygaZ	lmo1442	group_3413	lmo0910	group_11185	lmo0900	folB	folA
group_10972	lmo1452	group_3419	lmo0911	adeC	adeC	map_1	lmo1709
pcrB	lmo1760	gatB_4	gatB	dagK_3	lmo1753	group_45051	lmo1757
purD	purD	gatA_2	gatA	gltC_3	gltC	purB	purB

Table S4. Detailed information on MLST of *L. monocytogenes* strains isolated from beef.

Genbank	Strain	ST	CC	Lineage	Genbank	Strain	ST	CC	Lineage	Genbank	Strain	ST	CC	Lineage
GCA_003002735.1	CFSAN035200	9	CC9	II	GCA_030962365.1	L01071	31	CC31	II	GCA_022682455.1	LYJ24890	3	CC3	I
GCA_004667505.1	CFSAN048830	5	CC5	I	GCA_030962325.1	L01074	9	CC9	II	GCA_015525835.1	OSF107796	9	CC9	II
GCA_023573915.1	JW-2022B	321	CC321	II	GCA_030974325.1	L01075	29	CC29	II	GCA_015527105.1	OSF107798	8	CC8	II
GCA_023573925.1	JW-2022D	2	CC2	I	GCA_030965465.1	L01076	9	CC9	II	GCA_015527285.1	OSF107815	8	CC8	II
GCA_023574005.1	JW-2022E	155	CC155	II	GCA_030974305.1	L01078	5	CC5	I	GCA_015961325.1	OSF107894	121	CC121	II
GCA_023574035.1	JW-2022F	9	CC9	II	GCA_030970905.1	L01090	200	CC200	II	GCA_015961605.1	OSF107898	6	CC6	I
GCA_027980745.1	L17	9	CC9	II	GCA_030970825.1	L01091	37	CC37	II	GCA_015961305.1	OSF107908	6	CC6	I
GCA_027981075.1	L18	9	CC9	II	GCA_030970865.1	L01093	5	CC5	I	GCA_015961965.1	OSF107910	6	CC6	I
GCA_027982115.1	L22	9	CC9	II	GCA_030962305.1	L01096	9	CC9	II	GCA_015962185.1	OSF107914	1	CC1	I
GCA_027982085.1	L40	155	CC155	II	GCA_030970725.1	L01100	200	CC200	II	GCA_015961165.1	OSF107921	9	CC9	II
GCA_027982205.1	L41	155	CC155	II	GCA_022683845.1	L1106	9	CC9	II	GCA_015962575.1	OSF107924	91	CC14	II
GCA_027982145.1	L44	9	CC9	II	GCA_030961345.1	L01108	451	CC11	II	GCA_015963205.1	OSF107925	59	CC59	I
GCA_027982235.1	L53	9	CC9	II	GCA_030962245.1	L01112	580	CC9	II	GCA_015963165.1	OSF107930	1	CC1	I
GCA_030989305.1	L00363	9	CC9	II	GCA_030974205.1	L01115	5	CC5	II	GCA_015963025.1	OSF107934	1	CC1	I
GCA_030977345.1	L00369	120	CC8	II	GCA_030970685.1	L01116	200	CC200	II	GCA_015842075.1	OSF107943	9	CC9	II
GCA_030968765.1	L00372	120	CC8	II	GCA_030965425.1	L01120	236	CC121	II	GCA_015882185.1	OSF107953	1	CC1	I
GCA_030989225.1	L00389	14	CC14	II	GCA_030974245.1	L01122	200	CC200	II	GCA_015526265.1	OSF107984	-	-	-
GCA_030975385.1	L00407	121	CC121	II	GCA_030962225.1	L01123	37	CC37	II	GCA_015526905.1	OSF107989	-	-	-
GCA_030968705.1	L00412	616	CC6	I	GCA_030962205.1	L01128	9	CC9	II	GCA_015527225.1	OSF107990	-	-	-
GCA_030968685.1	L00417	415	CC11	II	GCA_030964725.1	L01132	101	CC101	II	GCA_015526285.1	OSF107995	9	CC9	II
GCA_030978765.1	L00431	59	CC59	I	GCA_030964665.1	L01135	54	CC54	I	GCA_015526605.1	OSF107996	-	-	-
GCA_022683605.1	L431	121	CC121	II	GCA_030973805.1	L01137	121	CC121	II	GCA_015526535.1	OSF107998	37	CC37	II
GCA_030975325.1	L00432	224	CC224	I	GCA_030964625.1	L01138	200	CC200	II	GCA_015526205.1	OSF107999	37	CC37	II
GCA_030978745.1	L00438	3	CC321	I	GCA_030970085.1	L01140	224	CC224	I	GCA_015526005.1	OSF108000	-	-	-
GCA_030978705.1	L00444	9	CC9	II	GCA_030969385.1	L01142	224	CC224	I	GCA_015533895.1	OSF108007	7	CC7	II
GCA_030978685.1	L00450	412	CC412	II	GCA_030969365.1	L01144	200	CC200	II	GCA_015525445.1	OSF108010	9	CC9	II
GCA_030977225.1	L00452	9	CC9	II	GCA_030969345.1	L01152	101	CC101	II	GCA_015535295.1	OSF108026	9	CC9	II
GCA_030978645.1	L00457	3	CC3	I	GCA_030973965.1	L01153	200	CC200	II	GCA_015564535.1	OSF108046	9	CC9	II
GCA_030977145.1	L00458	1	CC1	I	GCA_030964605.1	L01154	9	CC9	II	GCA_015525455.1	OSF108055	-	-	-
GCA_030975345.1	L00465	9	CC9	II	GCA_030964485.1	L01164	91	CC14	II	GCA_015534915.1	OSF108088	224	CC224	I
GCA_030975305.1	L00471	224	CC224	I	GCA_030960985.1	L01166	9	CC9	II	GCA_015535115.1	OSF108091	37	CC37	II
GCA_030968585.1	L00474	2	CC2	I	GCA_030973745.1	L01168	9	CC9	II	GCA_015534075.1	OSF108092	29	CC29	II
GCA_030977125.1	L00478	224	CC224	I	GCA_030961165.1	L01180	9	CC9	II	GCA_015533815.1	OSF108096	6	CC6	I
GCA_030968465.1	L00482	2	CC2	I	GCA_030970045.1	L01184	9	CC9	II	GCA_016448785.1	OSF108108	6	CC6	I
GCA_030982575.1	L00487	1	CC1	I	GCA_022683815.1	L1184	9	CC9	II	GCA_015539425.1	OSF108110	29	CC29	II
GCA_030977065.1	L00489	1	CC1	I	GCA_030961145.1	L01185	616	CC6	I	GCA_015561785.1	OSF108142	-	-	-
GCA_030978525.1	L00491	2	CC2	I	GCA_030964525.1	L01187	9	CC9	II	GCA_015536245.1	OSF108148	-	-	-
GCA_030978465.1	L00493	6	CC6	I	GCA_030964445.1	L01191	9	CC9	II	GCA_015535895.1	OSF108152	-	-	-
GCA_030975265.1	L00496	9	CC9	II	GCA_030961085.1	L01193	3217	CC736	I	GCA_015534475.1	OSF108172	224	CC224	I
GCA_030977025.1	L00508	9	CC9	II	GCA_030961105.1	L01194	9	CC9	II	GCA_015533545.1	OSF108183	6	CC6	I
GCA_030968405.1	L00533	580	CC9	II	GCA_030970025.1	L01199	9	CC9	II	GCA_015564835.1	OSF108193	1	CC1	I
GCA_030968345.1	L00545	9	CC9	II	GCA_030964385.1	L01219	3218	CC6	I	GCA_015563085.1	OSF108201	1	CC1	I
GCA_030975225.1	L00557	3214	CC14	II	GCA_030969945.1	L01223	37	CC37	II	GCA_015542345.1	OSF108217	37	CC37	II
GCA_030978405.1	L00574	3214	CC14	II	GCA_030969925.1	L01227	218	CC218	I	GCA_015563705.1	OSF108226	37	CC37	II
GCA_030976985.1	L00578	9	CC9	II	GCA_030975965.1	L01233	9	CC9	II	GCA_015535935.1	OSF108260	-	-	-
GCA_030976965.1	L00596	1	CC1	I	GCA_030975945.1	L01240	2	CC2	I	GCA_015534405.1	OSF108274	59	CC59	I
GCA_030968285.1	L00597	224	CC224	I	GCA_030973665.1	L01241	37	CC37	II	GCA_015531045.1	OSF108278	6	CC6	I
GCA_030978445.1	L00599	9	CC9	II	GCA_030972775.1	L01251	580	CC9	II	GCA_015533505.1	OSF108281	37	CC37	II
GCA_030978425.1	L00613	9	CC9	II	GCA_030969885.1	L01264	9	CC9	II	GCA_015564515.1	OSF108298	204	CC204	II
GCA_030975175.1	L00618	9	CC9	II	GCA_030969845.1	L01271	9	CC9	II	GCA_015539625.1	OSF108309	121	CC121	II
GCA_030968205.1	L00634	511	CC7	II	GCA_030972795.1	L01288	451	CC11	II	GCA_015565595.1	OSF108310	388	CC388	I
GCA_030975145.1	L00636	9	CC9	II	GCA_030960925.1	L01299	121	CC121	II	GCA_015542305.1	OSF108315	5	CC5	I
GCA_030968325.1	L00638	8	CC8	II	GCA_030972645.1	L01304	9	CC9	II	GCA_015561645.1	OSF108316	7	CC7	II
GCA_030978325.1	L00641	91	CC14	II	GCA_030972605.1	L01305	9	CC9	II	GCA_015564675.1	OSF108323	1	CC1	I
GCA_030978305.1	L00642	9	CC9	II	GCA_022683735.1	L1330	204	CC204	II	GCA_015563165.1	OSF108351	2	CC2	I
GCA_030976885.1	L00646	18	CC18	II	GCA_022683715.1	L1336	204	CC204	II	GCA_015562295.1	OSF108357	9	CC9	II
GCA_030978265.1	L00649	9	CC9	II	GCA_030964125.1	L01373	426	CC426	I	GCA_015565415.1	OSF108376	6	CC6	I
GCA_030968185.1	L00654	91	CC14	II	GCA_022683675.1	L1381	9	CC9	II	GCA_015564115.1	OSF108389	-	-	-
GCA_030967565.1	L00668	9	CC9	II	GCA_030964085.1	L01393	9	CC9	II	GCA_015565775.1	OSF108433	-	-	-

GCA_030967515.1	L00679	9	CC9	II	GCA_022683695.1	L1393	3	CC3	I	GCA_015963225.1	OSF108443	451	CC11	II
GCA_030978065.1	L00700	9	CC9	II	GCA_030960785.1	L01394	9	CC9	II	GCA_015584475.1	OSF108457	29	CC29	II
GCA_030976765.1	L00702	121	CC121	II	GCA_030972445.1	L01419	37	CC37	II	GCA_015583335.1	OSF108461	77	CC77	I
GCA_030967345.1	L00715	9	CC9	II	GCA_030960705.1	L01434	9	CC9	II	GCA_015584735.1	OSF108466	2	CC2	I
GCA_030989705.1	L00723	20	CC20	II	GCA_030969765.1	L01435	9	CC9	II	GCA_015962665.1	OSF108467	-	-	-
GCA_030967265.1	L00728	8	CC8	II	GCA_030975825.1	L01461	37	CC37	II	GCA_015599325.1	OSF108470	-	-	-
GCA_030967165.1	L00741	9	CC9	II	GCA_030960645.1	L01469	9	CC9	II	GCA_015595725.1	OSF108498	1296	CC8	II
GCA_030977925.1	L00745	9	CC9	II	GCA_030960585.1	L01470	124	CC124	II	GCA_015961665.1	OSF108569	3	CC3	I
GCA_030967065.1	L00752	7	CC7	II	GCA_030972365.1	L01486	379	CC379	I	GCA_015961585.1	OSF108570	6	CC6	I
GCA_030977885.1	L00762	18	CC18	II	GCA_030975805.1	L01488	2217	CC8	II	GCA_015961195.1	OSF108572	8	CC8	II
GCA_030974965.1	L00781	18	CC18	II	GCA_030963965.1	L01490	2	CC2	I	GCA_015962765.1	OSF108581	9	CC9	II
GCA_030966985.1	L00801	5	CC5	II	GCA_030960605.1	L01495	224	CC224	I	GCA_015583085.1	OSF108595	8	CC8	II
GCA_030976425.1	L00802	9	CC9	II	GCA_030963145.1	L01499	2	CC2	I	GCA_015583035.1	OSF108608	1	CC1	I
GCA_030977745.1	L00812	489	CC489	I	GCA_030960545.1	L01500	9	CC9	II	GCA_015842295.1	OSF108930	224	CC224	I
GCA_030966885.1	L00814	9	CC9	II	GCA_030975745.1	L01501	121	CC121	II	GCA_015841145.1	OSF108960	1	CC1	I
GCA_030974905.1	L00819	9	CC9	II	GCA_030963945.1	L01583	2217	CC8	II	GCA_015842055.1	OSF108966	416	CC1	I
GCA_030974925.1	L00823	37	CC37	II	GCA_030975765.1	L01584	6	CC6	I	GCA_015842415.1	OSF108976	9	CC9	II
GCA_030977665.1	L00825	5	CC5	I	GCA_030972235.1	L01587	9	CC9	II	GCA_018104005.1	OSF111252	3	CC3	I
GCA_022683035.1	L867	9	CC9	II	GCA_030963905.1	L01592	121	CC121	II	GCA_018103075.1	OSF111260	9	CC9	II
GCA_030977525.1	L00872	121	CC121	II	GCA_030969265.1	L01598	9	CC9	II	GCA_018103095.1	OSF111269	9	CC9	II
GCA_030976285.1	L00881	9	CC9	II	GCA_022684395.1	L1598	121	CC121	II	GCA_018103835.1	OSF111275	-	-	-
GCA_030962805.1	L00887	204	CC204	II	GCA_030963865.1	L01599	580	CC9	II	GCA_018103675.1	OSF111283	6	CC6	I
GCA_030962745.1	L00894	7	CC7	II	GCA_030960485.1	L01605	9	CC9	II	GCA_018104385.1	OSF111286	21	CC218	II
GCA_030963365.1	L00919	6	CC6	II	GCA_030969685.1	L01607	9	CC9	II	GCA_018103995.1	OSF111300	9	CC9	II
GCA_030974625.1	L00920	9	CC9	II	GCA_022684255.1	L1608	3	CC3	I	GCA_018103795.1	OSF111301	9	CC9	II
GCA_030976155.1	L00929	5	CC5	I	GCA_030963885.1	L01610	5	CC5	I	GCA_018103595.1	OSF111302	32	CC32	I
GCA_030974585.1	L00930	9	CC9	II	GCA_030960465.1	L01613	5	CC5	I	GCA_018104545.1	OSF111307	6	CC6	I
GCA_030976085.1	L00936	59	CC59	I	GCA_030960425.1	L01617	9	CC9	II	GCA_018104155.1	OSF111308	-	-	-
GCA_030974545.1	L00941	8	CC8	II	GCA_030963845.1	L01619	9	CC9	II	GCA_020803475.1	PNUSAL012100	271	CC177	II
GCA_030969505.1	L00943	77	CC77	I	GCA_030963645.1	L01621	5	CC5	I	GCA_020803995.1	PNUSAL012101	271	CC177	II
GCA_030962605.1	L00946	9	CC9	II	GCA_030963625.1	L01646	5	CC5	I	GCA_020804135.1	PNUSAL012102	271	CC177	II
GCA_030962525.1	L00947	9	CC9	II	GCA_022684535.1	L1661	321	CC321	II	GCA_020803755.1	PNUSAL012103	5	CC5	I
GCA_030971885.1	L00958	5	C5	I	GCA_030963565.1	L01716	451	CC11	II	GCA_020804155.1	PNUSAL012104	5	CC5	I
GCA_030962505.1	L00959	1	CC1	I	GCA_030963545.1	L01733	14	CC14	II	GCA_021528855.1	PNUSAL012900	9	CC9	II
GCA_030966305.1	L00960	9	CC9	II	GCA_030975725.1	L01750	5	CC5	I	GCA_021546375.1	PNUSAL012909	9	CC9	II
GCA_030976045.1	L00973	1	CC1	I	GCA_022684495.1	L1756	204	CC204	II	GCA_021546295.1	PNUSAL012910	5	CC5	I
GCA_030971865.1	L00981	321	CC321	II	GCA_030960405.1	L01771	3220	CC9	II	GCA_022628835.1	PNUSAL013433	199	CC199	II
GCA_030974565.1	L00982	321	CC321	II	GCA_022684215.1	L1778	204	CC204	II	GCA_023270935.1	PNUSAL013434	5	CC5	I
GCA_030971745.1	L00988	321	CC321	II	GCA_030975665.1	L01795	451	CC11	II	GCA_022657505.1	PNUSAL013435	321	CC321	II
GCA_030965605.1	L01004	9	CC9	II	GCA_030963105.1	L01799	121	CC121	II	GCA_022657475.1	PNUSAL013436	1106	CC5	I
GCA_030961485.1	L01009	451	CC11	II	GCA_030975645.1	L01800	451	CC11	II	GCA_022629855.1	PNUSAL013437	9	CC9	II
GCA_030971695.1	L01012	321	CC321	II	GCA_030963505.1	L01810	9	CC9	II	GCA_022657495.1	PNUSAL013438	5	CC5	I
GCA_030965545.1	L01019	236	CC121	II	GCA_030969665.1	L01835	9	CC9	II	GCA_022629015.1	PNUSAL013439	5	CC5	I
GCA_030974505.1	L01023	580	CC9	II	GCA_030975605.1	L01851	9	CC9	II	GCA_024849965.1	PNUSAL014862	9	CC9	II
GCA_030962465.1	L01025	580	CC9	II	GCA_022684225.1	L1861	321	CC321	II	GCA_024849945.1	PNUSAL014863	9	CC9	II
GCA_030962445.1	L01026	616	CC6	I	GCA_022684175.1	L2099	-	-	-	GCA_024850005.1	PNUSAL014864	1106	CC5	I
GCA_030965515.1	L01030	9	CC9	II	GCA_022684155.1	L2119	155	CC155	II	GCA_025310455.1	PNUSAL015301	5	CC5	I
GCA_030963405.1	L01038	5	CC5	I	GCA_022684065.1	L2124	155	CC155	II	GCA_025264765.1	PNUSAL015302	5	CC5	I
GCA_030974435.1	L01041	9	CC9	II	GCA_022684115.1	L2128	155	CC155	II	GCA_025311335.1	PNUSAL015303	-	-	-
GCA_030962405.1	L01042	9	CC9	II	GCA_022684135.1	L2131	155	CC155	II	GCA_025413215.1	PNUSAL015360	5	CC5	I
GCA_030974425.1	L01045	37	CC37	II	GCA_022684315.1	L2132	155	CC155	II	GCA_025415875.1	PNUSAL015361	5	CC5	I
GCA_030974405.1	L01049	3	CC3	I	GCA_022684295.1	L2146	204	CC204	II	GCA_025414895.1	PNUSAL015362	5	CC5	I
GCA_030974385.1	L01050	2	CC2	I	GCA_022684445.1	L2185	204	CC204	II	GCA_025412545.1	PNUSAL015363	5	CC5	I
GCA_030963415.1	L01053	398	CC19	II	GCA_003191365.1	LM-F-24	155	CC155	II	GCA_040551615.1	PNUSAL022259	5	CC5	I
GCA_030971115.1	L01054	6	CC6	I	GCA_003191285.1	LM-F-25	5	CC5	I	GCA_013415135.1	SCPM-O-B-8838	451	CC11	II
GCA_030971055.1	L01065	9	CC9	II	GCA_003190965.1	LM-F-31	120	CC8	II					
